# The biocontrol endophytic bacterium *Pseudomonas fluorescens* PICF7 induces systemic defense responses in aerial tissues upon colonization of olive roots

**DOI:** 10.3389/fmicb.2014.00427

**Published:** 2014-09-05

**Authors:** Carmen Gómez-Lama Cabanás, Elisabetta Schilirò, Antonio Valverde-Corredor, Jesús Mercado-Blanco

**Affiliations:** Lab Plant-Microbe Interactions, Department of Crop Protection, Institute for Sustainable Agriculture, Agencia Estatal Consejo Superior de Investigaciones Científicas (CSIC)Córdoba, Spain

**Keywords:** *Olea europea*, biological control, endophyte, *Pseudomonas fluorescens*, systemic defense response, qRT-PCR, Verticillium wilt

## Abstract

*Pseudomonas fluorescens* PICF7, a native olive root endophyte and effective biocontrol agent (BCA) against Verticillium wilt of olive, is able to trigger a broad range of defense responses in root tissues of this woody plant. In order to elucidate whether strain PICF7 also induces systemic defense responses in above-ground organs, aerial tissues of olive plants grown under non-gnotobiotic conditions were collected at different time points after root bacterization with this endophytic BCA. A suppression subtractive hybridization (SSH) cDNA library, enriched in up-regulated genes, was generated. This strategy enabled the identification of 376 ESTs (99 contigs and 277 singlets), many of them related to response to different stresses. Five ESTs, involved in defense responses, were selected to carry out time-course quantitative real-time PCR (qRT-PCR) experiments aiming to: (1) validate the induction of these genes, and (2) shed light on their expression pattern along time (from 1 to 15 days). Induction of olive genes potentially coding for lipoxygenase 2, catalase, 1-aminocyclopropane-1-carboxylate oxidase, and phenylananine ammonia-lyase was thus confirmed at some time points. Computational analysis also revealed that different transcription factors were up-regulated in olive aerial tissues (i.e., JERF, bHLH, WRKY), as previously reported for roots. Results confirmed that root colonization by this endophytic bacterium does not only trigger defense responses in this organ but also mounts a wide array of systemic defense responses in distant tissues (stems, leaves). This sheds light on how olive plants respond to the “non-hostile” colonization by a bacterial endophyte and how induced defense response can contribute to the biocontrol activity of strain PICF7.

## Introduction

Olive (*Olea europaea* L.) is an emblematic woody plant in the Mediterranean Basin. Its cultivation for millennia has shaped a characteristic agro-ecosystem in the region, representing a traditional agricultural activity with indisputable social, economical, and historical relevance (Caballero and del Río, 2008). From the original domestication and cultivation area, olive cropping is expanding to other climatically-favorable geographical areas around the globe (Barranco et al., 2008). Moreover, consumption of olive oil, the major product extracted from olive drupe, is gaining interest because of the increasing body of knowledge showing its beneficial effects for human health compared to other fat diet (Pauwels, 2011). The traditional landscape of olive orchards found throughout the Mediterranean Basin is progressively changing toward more productive cropping systems with high tree densities (up to 2000 trees/ha) (Navarro and Parra, 2008; Rallo et al., 2012; Connor et al., 2014). These changes lead to an increasing demand for nursery-produced planting material that must be pathogen-free certified and/or protected against pathogen attacks (Tjamos, 1993; López-Escudero and Mercado-Blanco, 2011).

Among the biotic constraints affecting olive cultivation, the soil-borne fungus *Verticillium dahliae* Kleb., causal agent of Verticillium wilt of olive (VWO), is considered the most threatening disease in many areas where this tree is cultivated. During the last two decades, the incidence of VWO has increased considerably. This could be due to factors such as (i) the use of pathogen-infested soil or infected propagation material, (ii) the pathogen's dispersal efficacy, (iii) the abuse on fertilization and irrigation, (iv) variable edaphic and climatic factors, (v) the high genetic/pathogenic diversity found within pathogen populations (i.e., defoliating [D] and non-defoliating [ND] pathotypes), or (vi) the changes in cultivation systems (López-Escudero and Mercado-Blanco, 2011). These factors, among others, makes VWO very difficult to control. Thus, the current spread of the disease and the severity of its attacks can only be effectively confronted by implementing an integrated disease management strategy (López-Escudero and Mercado-Blanco, 2011). A promising control tool within this strategy is the use of microbial antagonists effective against *V. dahliae*, particularly at the nursery stage (Mercado-Blanco et al., 2004).

Some plant growth promoting rhizobacteria (PGPR) are able to protect their hosts against phytopathogens displaying biocontrol activity (Van Loon, 2007; Beneduzi et al., 2012). This positive effect can be exerted through different mechanisms such as root architecture modification (Vacheron et al., 2013), production of antibiotics (Beneduzi et al., 2012), and/or triggering a specific defense signaling pathway known as induced systemic resistance (ISR) (Bakker et al., 2007). The possibility to exploit ISR within integrated control strategies has been proposed elsewhere (Ramamoorthy et al., 2001; Zehnder et al., 2001). ISR is phenotypically similar to systemic acquired resistance (SAR), a response activated, for instance, after a first infection by an incompatible necrotizing pathogen (Durrant and Dong, 2004). Nevertheless, the signal transduction pathway and the molecular basis underlying both responses are different. In SAR, defense reactions such as hypersensitive response (HR), salicylic acid (SA) biosynthesis or induction of pathogenesis-related (PR) proteins are characteristic (Andreu et al., 2006). In contrast, ISR depends on pathways regulated by the plant hormones ethylene (ET) and jasmonic acid (JA), and include the induction of enzymatic activities related to phenylalanine ammonia-lyase (PAL) synthesis, among others (Sena et al., 2013). Both pathogenic and beneficial bacteria can cause responses such as plant cell wall reinforcement and production of phytoalexins and PR proteins, consistent with the fact that SAR and ISR represent an enhanced basal plant resistance, and that plant hormone(s)-mediated cross talk is taking place between both mechanisms (Pieterse et al., 2009, 2012).

One of the most promising biocontrol agents (BCA) so far studied for the biocontrol of VWO is *Pseudomonas fluorescens* PICF7. Strain PICF7 is a indigenous colonizer of olive roots, an *in vitro* antagonist of *V. dahliae*, and an effective BCA against VWO caused by the D pathotype in young, nursery-produced olive plants (Mercado-Blanco et al., 2004; Prieto et al., 2009). Our previous works have also demonstrated that PICF7 is able to endophytically colonize olive root tissues under different experimental conditions (Prieto and Mercado-Blanco, 2008; Prieto et al., 2009, 2011). Bacterial endophytes, likely present in all plant species (Rosenblueth and Martínez-Romero, 2006; Hardoim et al., 2008; Reinhold-Hurek and Hurek, 2011), offer an interesting potential to be used in agricultural biotechnology. They can exert plant growth promotion and biocontrol activity and, in addition, are adapted to the ecological niche (plant tissues) where their beneficial effects can be deployed (Mercado-Blanco and Lugtenberg, 2014). Living inside the plant means that endophytes must be able to counteract defense responses deployed by the host against their colonization. This scenario likely implies an as yet poorly-understood modulation of the plant immune response enabling endophytes to be recognized and tolerated by the host plant as “friendly” invasors (Reinhold-Hurek and Hurek, 2011; Zamioudis and Pieterse, 2012; Mercado-Blanco and Lugtenberg, 2014).

Our knowledge on the genetic basis underlying the interaction between endophytes and their hosts is still very limited (Bordiec et al., 2010; Reinhold-Hurek and Hurek, 2011). Nevertheless, some studies have shown that inner colonization of plant tissues by bacterial endophytes triggers, among other changes, a wide range of defense responses (Wang et al., 2005; Conn et al., 2008). Our previous works have demonstrated that colonization by *P. fluorescens* PICF7 induced a broad set of defense responses in olive root tissues, including genes related to ISR and SAR (Schilirò et al., 2012). Indeed, root colonization of “Arbequina” plants by this BCA produced the differential expression of genes involved in processes such as plant hormones and phenylpropanoids biosynthesis and PR proteins, as well as the up regulation of several transcription factors implicated in systemic defensive responses (WRKY5, bHLH, ARF2, GRAS1) (Schilirò et al., 2012).

In this study, we aimed to elucidate whether systemic defense responses are also triggered in aerial olive tissues upon root inoculation with strain PICF7. We also aimed to figure out whether these responses are similar to the transcriptional changes observed in roots during the interaction with this endophyte. Whereas, our main objective was to unravel the broad genetic changes taking place in above-ground tissues, we also focused on the time-course expression of specific defense genes. Thus, expression of genes potentially coding for olive lipoxygenase (*LOX-2*), phenylalanine ammonia lyase (*PAL*), acetone cyanohydrin lyase (*ACL*), all of them previously reported to be induced in olive roots upon PICF7 inoculation, as well as a catalase (*CAT*), and 1-aminocyclopropane-1-carboxylate oxidase (*ACO*), was studied. Results showed that strain PICF7 was able to trigger a wide array of systemic defense responses in aerial olive tissues, some of them being previously reported to be induced in roots. This points out to the ability of PICF7 to elicit broad transcriptional changes, mostly of defensive nature, at distant parts of the plant. Remarkably, the genetic changes here reported have been unraveled in a plant-microbe interaction poorly investigated so far; that is, between a woody plant of commercial interest (olive) and an effective biocontrol endophytic bacterium. Furthermore, we have implemented a non-gnotobiotic experimental set-up, a more natural scenario not frequently used in this type of studies.

## Materials and methods

### Plant material, *Pseudomonas fluorescens* PICF7 root treatment, and mRNA purification

Aerial olive tissues used in this study originated from plants used in an experimental set-up described by Schilirò et al. (2012). Olive plants (cv. Arbequina, 3-month-old) were propagated in a commercial nursery located in Córdoba province (Southern Spain). Prior to bacterial treatment, plants were acclimated for three weeks in a growth chamber under conditions described below. Inoculum of strain PICF7 (Mercado-Blanco et al., 2004) was prepared as described by Prieto and Mercado-Blanco (2008). “Arbequina” plants were manipulated and their root systems bacterized in a suspension of PICF7 cells (15 min, 1·10^8^ cells ml^−1^) as previously described (Schilirò et al., 2012). Roots of control plants (non-bacterized) were dipped in 10 mM MgSO_4_·7 H_2_O. Then, plants were individually transplanted into polypropylene pots containing an autoclaved sandy substrate (Prieto and Mercado-Blanco, 2008). Plants were maintained at controlled conditions (23 ± 1°C, 60–90% relative humidity, 14-h photoperiod of fluorescent light at 360 μE m^−2^) during 21 days (Schilirò et al., 2012). In order to alleviate stress of plants after manipulation, inoculation, and transplanting, the above-mentioned photoperiod was enlarged progressively until reaching 14-h daylight.

To obtain a broad range of differentially-expressed (induced) genes, the whole aerial part (stems plus leaves) of each olive plant was sampled at different times after treatments. Therefore, aerial tissues were collected at 0 h, 5 h, 10 h, 24 h and 2, 3, 4, 7, 10, 12, 15, 18, and 21 days (two plants/time point) for both inoculated (bacterized) and non-inoculated (control) plants. Aerial tissues of 52 plants (26 bacterized and 26 control) were sampled, rapidly frozen using liquid nitrogen, and stored at −80°C until used. Total RNA of each sample was extracted according to Asif et al. (2000). The removal of contaminating genomic DNA was carried out by DNaseI treatment (Roche Applied Science, Mannheim, Germany) according to the manufacturer's instructions. All RNA samples corresponding to each treatment (bacterized and non-bacterized plants) were pooled separately to obtain two independent RNA pools prior to mRNA purification. Poly A+ mRNA was purified from approximately 400 μg of total RNA of each pool using the Dynabeads® mRNA Purification Kit (Dynal Biotech, Oslo, Norway) according to the manufacturer's indications. Purity and quality of mRNA samples were verified by both agarose gel electrophoresis and spectrophotometry using a ND-1000 Spectrophotometer (NanoDrop Technologies, Wilmington, DE).

### Generation of a “Suppression Substractive Hybridization” cDNA library enriched in induced genes of olive aerial tissues, cloning and sequencing

A cDNA library was constructed by using the “Suppression Subtractive Hybridization” (SSH) technology (Diatchenko et al., 1996) in order to clone and identify genes up-regulated in aerial tissues during the interaction of *P. fluorescens* PICF7 with olive roots. SSH allows enrichment and cloning of less abundant transcripts through amplification and normalization of subtracted cDNAs. The cDNA library was generated using the PCR-Select™ cDNA Subtraction Kit (BD Biosciences, Palo Alto, CA) according to the manufacturer's instructions and as previously described by Schilirò et al. (2012). Briefly, cDNAs were separately synthesized from 2 μg of mRNA of each PICF7-inoculated (tester) and control (driver) plant, digested with *RsaI* and ligated to adaptors 1 and 2R. To enrich differentially-expressed sequences, two rounds of hybridization and PCR amplification were carried out. Advantage® 2 PCR Kit (BD Biosciences) was used for PCR amplifications in total volume of 50 μL. The amplification program was: denaturation for 5 min at 94°C, followed by 33 cycles of 30 s at 94°C, 45 s at 55°C and 1 min at 72°C and a final extension step of 10 min at 72°C. To check the efficiency of subtraction a 308-bp fragment was amplified using the primer pair Act1-fw: 5′-GCTTGCTTATGTTGCTCTCGAC-3′/Act1-rv: 5′-TGATTTCCTTGCTCATACGGTC-3′) belonging to the constitutively expressed (housekeeping) β-actin gene from olive (Acc. No. AF545569) (Table 1), whose expression was checked not to be influenced by strain PICF7 inoculation (Schilirò et al., 2012).

**Table 1:** **List of selected transcripts induced in olive aerial tissues upon *P. fluorescens* PICF7 colonization used in qRT-PCR experiments**.

**Clone ID**	**Putative gene**	**Process**	**Primer pair**	**Amplicon length (bp)**	**Linear equation (1)**	**R^2^(1)**	**PCR efficiency(1)**
AU12-D10T7	1-Aminocyclopropane-1-carboxylate oxidase	Ethylene biosynthesis	Fw: CTCAAGTTGATCCCCAAT	231	*Y* = −3.430 + 29.271	0.994	95.7
			Rv: GCATTCCATGGCTCTAAA				
AU03-E02T7	Phenylananine ammonia-lyase	Phenylpropanoids biosynthesis	Fw: ACATAGGAGGACCAAACAA	280	*Y* = −3.394 + 29.944	0.991	97.1
			Rv: GTAGGATAAAGGGACAAGAT				
AU06-H07T7	Catalase	Oxidation-reduction	Fw: CCCAGGATCTCTACGATT	273	*Y* = −3.371 + 22.018	0.993	98.0
			Rv: TCTGGAGCATCTTGTCAT				
AU13-A07T7	Lipoxygenase 2	Linolenic acid metabolism	Fw: GAGACATCACCAATCCAT	141	*Y* = −3.413 + 29.253	0.989	96.3
			Rv: CCAGCACCACATCTATTT				
AU04-G10T7	Acetone cyanohydrin lyase	Salicylic acid-binding protein 2	Fw: GAAAGAGATGGAGACGGAA	246	*Y* = −3.141 + 32.360	0.994	108.2
			Rv: ACACAGGGAAATGCATCAAA				
AF545569 (2)	*Olea europea* beta-actin (act1)	Cytoskeletal integrity	Fw: GCTTGCTTATGTTGCTCTCGAC	308	*Y* = −3.341 + 28.428	0.990	99.2
			Rv: TGATTTCCTTGCTCATACGGTC				

For all transcripts, relative expression (RE) analysis was repeated at least two times in independent qRT-PCR experiments (see text and **Figure 4**). Clone ID, gene name, biological process, primers sequences, amplicon length, linear equations, correlation coefficients (R^2^), and PCR efficiencies are indicated (Fw: Forward; Rv: Reverse). AU, Arbequina aerial tissues induced gene; (1) Linear equation R^2^ coefficient and PCR efficiency correspond to a representative qRT-PCR assay performed (2): olive act-1 gene used as reference to normalize relative expression (Gene Bank Accession Number). doi: 10.1371/journal.pone.0048646.t003.

Products resulting from SSH (cDNAs) were ligated in the pGEM-T Easy Vector (Promega, Madison, WI) and cloned into *Escherichia coli* CH3-Blue competent cells (Bioline, London, UK) according to manufacturer's specifications. Positive colonies based on white/blue color selection were grown in 96-well microtiter plates contained LB medium with 100 mg L^−1^ and were incubated at 37°C for 22 h. Lastly, forward T7 universal primer was employed to isolate and sequence 1344 bacterial clones from the SSH library. Sequencing of DNAs was performed at a commercial service (Sistemas Genómicos S.L., Valencia, Spain).

### Bioinformatics analysis of expressed sequence tags

Contaminating vector and adaptors sequences were identified and eliminated from each Expressed Sequence Tags (ESTs) by mass alignment using the “CLC Main Workbench 6.8.1” (CLC bio, Aarhus, Denmark) software. Sequences showing low quality or length (<100 bp) were excluded from the analysis (“sequence trimming” step). The “CLC Main Workbench 6.8.1” software was used to assemble the EST data set, aiming to find contiguous sequences and redundancy. Computational annotation of ESTs obtained during olive-PICF7 interaction was carried out by using the open software “Blast2GO version 2.7.0” (Conesa et al., 2005) available at http://www.blast2go.com/b2ghome. Homologies were checked in the non-redundant (nr) GenBank protein database by running the Blastx algorithm (Altschul et al., 1990) with the *E*-value set to 1.0E-3 and the High-scoring Segment Pairs (HSP) length cutoff fixed to 33 (as previously implemented by Schilirò et al., 2012).

The “Blast2GO software v.2.7.0” was used to perform Gene Ontology (GO) analysis from retrieved database matches. Functional annotation of all sequences was carried out using default parameters. InterPro Scan (Zdobnov and Apweiler, 2001) was used to associate functional information and GO terms to the protein of interest by using the specific tool implemented in the Blast2GO software with the default parameters. Finally, the “Augment Annotation by ANNEX” function was used to improve the annotation profiles information. The GOslim “goslim_plant.obo” was run to achieve specific plant GO terms by means of a plant-specific reduced version of the GO (available at http://www.arabidopsis.org/). Enzyme mapping of annotated sequences was retrieved by direct GO to Enzyme annotation and used to query the Kyoto Encyclopedia of Genes and Genomes (KEGG—http://www.genome.jp/kegg/) to define the main metabolic pathways involved. The distribution of hits obtained against entries for other plants within the NCBI database was used to get a descriptive view of the newly generated dataset.

### Data validation and time-course gene expression profile

A selection of up-regulated ESTs identified by the Blast2GO tool was used for validation by quantitative real-time PCR (qRT-PCR) experiments. Five transcripts from the whole dataset of nr sequences found to be up-regulated and belonging to key biosynthetic and metabolic pathways were chosen (*LOX-2, PAL, ACL, CAT*, and *ACO*). Moreover, these ESTs fulfilled the criteria of >100-bp long and *E*-value ≤ 1.0E-3 Selected genes were tested by qRT-PCR at four different times to assess their expression patterns in above-ground organs at different time points after inoculation of roots with strain PICF7. Specific primer pairs for these five sequences (Table 1) were designed using the “CLC Main Workbench 6.8.1” (CLC bio) software and tested for their specificity in a temperature range (53 to 63°C) by conventional PCR. To find the appropriate range of concentrations at which target cDNA, specific qRT-PCR assays were conducted using cDNAs synthesized from 10-fold serially diluted (1 μg, 100 ng, 10 ng, 1 ng, 100 pg) RNA samples. Standard curves were generated for each selected transcript using reverse transcribed cDNA from serial dilutions (300 ng, 30 ng, 3 ng, 0.3 ng) of remnant samples of total RNA not used for SSH and that were properly stored at −20°C. Gene expression of selected genes was measured at four different times: 1, 3, 7, and 15 days after inoculation (DAI) with PICF7. Ct values and the logarithm of cDNA concentrations were linearly correlated for each of the examined genes and PCR efficiencies were calculated by iQ5 optical system software v.2.1 (BioRad, Hercules, CA). Synthesis of cDNA was performed using the “iScript cDNA Synthesis Kit” (BioRad) from 100 ng of total RNA in each of four different times assayed, and following the manufacturer's procedure. qRT-PCR experiments and analyses were done in a thermal cycler iQ5 Real-Time PCR System (BioRad) provided with a 96-well sample block. For each selected gene, expression was quantified at least two times in independent qRT-PCR experiments, and three replicas per point studied and per plate were routinely included.

All qRT-PCR reactions were performed containing 2 μL of cDNA, 0.5 μM of each primer, 10 μL of 2 × iQ™ SYBRH Green Supermix (BioRad) and H_2_O up to a total volume of 20 μL. The following parameters were used in all reactions: 94°C for 5 min, 50 cycles of 94°C for 30 s, 55°C for 30 s, and 72°C for 40 s. Linear equations, correlation coefficients (*R*^2^) and reaction efficiencies were estimated for each transcript. Melting curves of qRT-PCR products were assessed from 55 to 95°C to confirm the amplification of single PCR bands. For all samples reaction protocol was as follows: 5 min at 95°C for initial denaturation, cooling to 55°C and melting from 55 to 95°C with a 0.5°C transition rate every 10 s.

The *O. europaea* β-actin gene was used as housekeeping gene to normalize data obtained from qRT-PCR assay and was amplified in the same conditions above described. Relative expression (RE) levels at different times were calculated according to Livak and Schmittgen (2001). The average of each expression gene fold change was categorized as follows: “low” ≥–1.0 to ≤1.0; “medium” ≥–2.0 to <–1.0 or >1.0 to ≤2.0; “high,” <–2.0 or >2.0 (Kim et al., 2008). All relative expression data in four different times for each gene were represented in a graphic as means ± STD of at least two separate experiments, each performed with triplicate samples. A paired sample *T*-test was performed to determine whether there was significant difference between the average values of each relative gene expression independent experiment (between plates). For all genes tested there was no significant difference between experiments (*P* > 0.05). *T*-test analysis was performed using the Statistix software (Version 9.0 for Windows).

### Accession numbers

ESTs reported in this study have been deposited in the dbESTs database of the National Center for Biotechnology Information (NCBI) under GenBank accession numbers JZ534362 (dbEST_Id 78897695)—JZ534925 (dbEST_Id 78898258).

## Results

### Construction and characterization of a cDNA library of olive genes induced in aerial tissues upon colonization of roots by *P. fluorescens* PICF7

A cDNA library enriched in olive transcripts up-regulated in aerial tissues after root inoculation with the biocontrol endophytic strain PICF7 was generated. A total number of 1344 ESTs were sequenced. Eventually, ESTs in the cDNA library were assembled into 99 distinct contigs (average length of 443 bp) and 277 singlets (average length of 338 bp) to provide a set of 376 unigenes differentially expressed (induced) in above-ground organs during PICF7-olive roots interaction. Despite the fact that the number of ESTs sequenced in this study was higher than that from root tissues (904 ESTs, Schilirò et al., 2012), the number of up-regulated unigenes found in aerial tissues was lower than in roots (445 ESTs, Schilirò et al., 2012).

**Figure 1:**
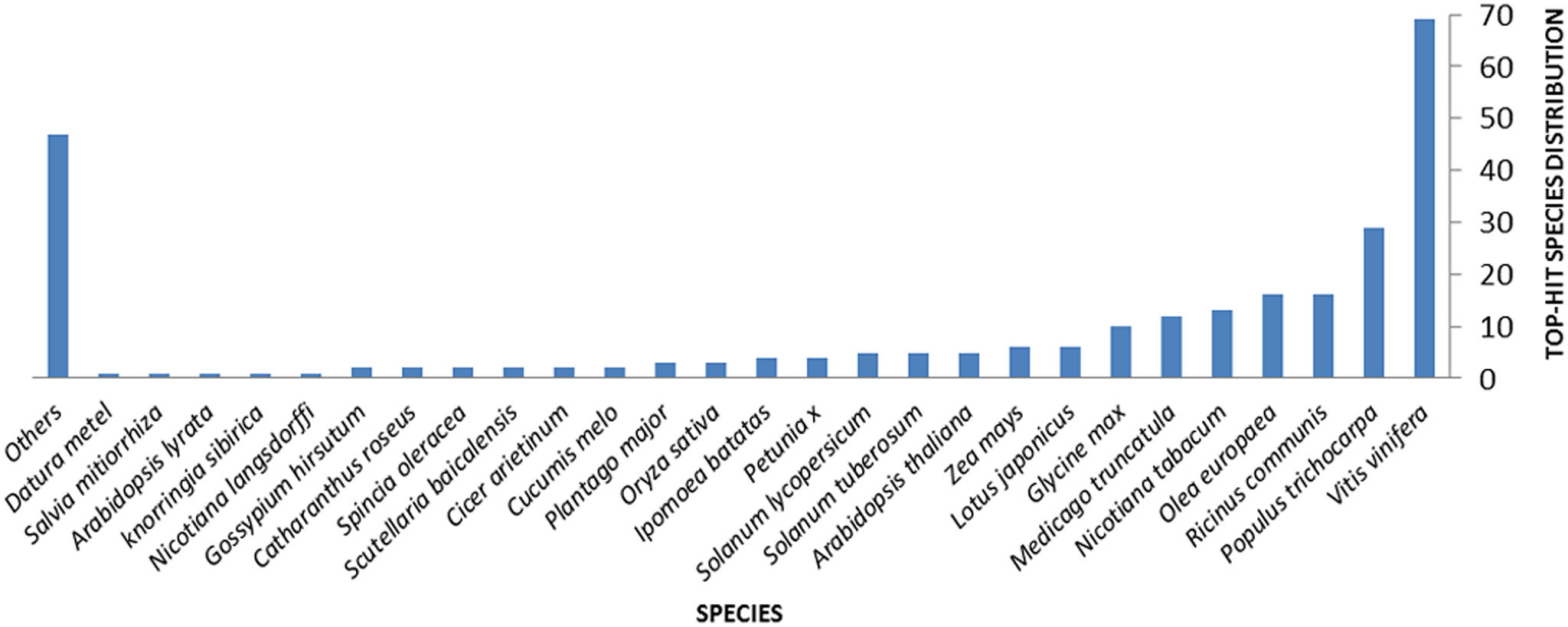
**Distribution of hits of the complete assembly obtained against sequences available in Uniprot for different plant species**.

Querying (Blastx) the nr NCBI database allowed the attribution of homologous hits for 71.8% of the ESTs. Hits distribution of the complete EST set against sequences from different plant species are shown in Figure 1. In particular, 130 ESTs (34.6% of the whole ESTs set) correspond to coding sequences previously identified in genomes of woody plants such as grape vine (*Vitis vinifera* L., 69 hits), western balsam poplar (*Populus trichocarpa* Torr. and A.Gray, 29 hits), castor bean (*Ricinus communis* L., 16 hits), and olive (16 hits) (Figure 1 and Table 2). *E*-values for this homology analysis ranged from 5.02E-3 to 1.06E-139. Only 4.3% of the 376 unigenes showed significant homology with olive genes in the databases [NCBI dbEST (http://www.ncbi.nlm.nih.gov/dbEST/)] (Table 2), indicating an as yet important lack of genetic/genomic information for this relevant woody crop (last search perfomed on May 2014). Finally, a total of 106 unigenes (28.2% of induced transcripts found in this work) were of unknown function.

**Table 2:** **List of induced EST sequences identified after Blastx analysis as homologous to olive (*Olea europaea* L.) genes previously indexed in databases**.

**EST sequence name**	**Putative protein function**	**Accession number**	***E*-Value**
AU01-B01T7	protein	AF28695	2,6E-47
AU01-D04T7	beta-glucosidase g1	AAL93619	2,70E-61
AU02-A03T7	low-temperatura inducible	ABS72020	1,93E-60
AU02-A05T7	beta-glucosidase d4	ACD43481	6,73E-55
AU02-C12T7	ribulose bisphosphate carboxylase oxygenase activase chloroplastic-like	ABS72022	5,37E-23
AU02-E06T7	chloroplast ribulose- -bisphosphate carboxylase oxygenase small subunit	ABS71998	1,93E-12
AU03-F12T7	psa1 protein	ABU39903	4,26E-48
AU04-G06T7	thaumatin-like protein	ACZ57583	5,98E-26
AU05-F08T7	1-deoxy-d-xylulose 5-phosphate reductoisomerase	AFS28671	3,75E-59
AU08-E04T7	cytochrome p450	AFS28690	8,09E-63
AU11-A01T7	auxin-induced protein pcnt115	ABS72001	2,29E-15
AU13-A01T7	loxc homolog	ADC43485	1,02E-61
AU13-A07T7	lipoxygenase	ADC43484	8,73E-25
AU13-E01T7	serine hydroxymethyltransferase	ABS72016	1,60E-86
AU-C1	bark storage protein a-like	AFP49328	1,95E-120
AU-C172	calcium-binding protein cml27	Q9M7R0	4,58E-61

Analysis of the 376 olive ESTs showed that many of the ESTs identified as induced in aerial olive tissues during the interaction of PICF7 in roots were related with plant responses to different stimuli and stresses (biotic, abiotic, endogenous, extracellular, or/and external). For instance, genes potentially coding for a 14-3-3-protein [EST sequence name AU11-F06] (response to stress), CAT [AU03-B01] (response to stress and abiotic, extracellular and endogenous stimuli), ACO [AU12-D10], phosphatase 2c [AU02-A06], and glycine rich RNA binding [AU01-H12] (response to stress, abiotic, biotic, and endogenous stimuli), PAL [AU03-E02] and ACL [AU04-G10] (response to pathogens), LOXs [AU13-A01; AU13-A07] (response to stress, biotic, and abiotic stimuli), were found to be up-regulated. Some of the ESTs were different kind of calcium (Ca^2+^)-binding proteins that could be related with plant defense reaction, e.g., Ca^2+^-binding protein cm127 [AU-C172], calreticulin [AU02-C02], calreticulin-3 parcial [AU07-B01], calmodulin (CaM) [AU-C96], and ef hand family protein [AU01-G11]. A complete list of ESTs identified as induced in above-ground organs after inoculation of olive roots with *P. fluorescens* PICF7 is shown in Table S1 as supplementary information for the reader. In addition, Table S2 displays contigs identified with their corresponding contiguous/overlapping ESTs. ESTs with unknown functions were not included in these tables. Results revealed that only a small percentage of induced unigenes (26 out of 376, representing 7.18% of all transcripts) were found in both the cDNA libray of aerial tissues (this study) and that one previously reported for root tissues (Schilirò et al., 2012) (Table 3). In particular, 4 out of the 26 putative proteins shared the same accession number when a blastx analysis was performed: serine protease (CAA07250), CAT (CAB56850), cyclophilin (ABS30424), and ef hand family protein (XP_002319225). Three of these proteins seem to be related to defensive response in plants. Thus, CAT and ef hand family protein take part in Ca^2+^ metabolism, biotic and abiotic stress (Day et al., 2002; Yang and Poovaiah, 2002). On the other hand, cyclophilin belongs to a family of immunosuppressant receptors called immunophilins that is expressed during pathogenic infection and abiotic stress condition (Romano et al., 2004).

**Table 3:** **Putative protein functions identified in *Pseudomonas fluorescens* PICF7-induced EST sequences in both roots and aerial tissues of olive (*Olea europaea* L.)**.

**Putative protein function**	**EST name sequence (root)**	**Accession number (root)**	**EST name sequence (aerial tissues)**	**Accesion number (aerial tissues)**
blight-associated protein p12	ARBRI-4_T7_B03	EAZ09461	AU02-F12T7	ADG29118
phenylalanine ammonia-lyase	ARBRI-C73	BAA95629	AU02-E02T7	ABS58596
Catalase	ARBRI-C50,	AAC19397, ABM47415,	AU03-B01T7, AU06-H07T7	CBA13361,
	ARBRI-2_T7_C02,	NP_001048861,		CAB56850^*^
	ARBRI-2_T7_C12,	CAB56850^*^		
	ARBRI-10_T7_B01			
glutamate decarboxylase	ARBRI-6_T7_E02	XP_002528515	AU04-A03T7	XP_003538378
thaumatin-like protein	ARBRI-C25	AAK59275	AU04-G06T7	ACZ57583
acetone cyanohydrin lyase	ARBRI-C140	AAR87711	AU04-G10T7	Q6RYA0
cytochrome p450	ARBRI-C12, ARBRI-C53,	P93531, ABC68413,	AU09-G05T7, AU08-E04T7,	XP_003610581,
	ARBRI-C103,	CBI30225, CBI19610	AU02-H09T7	AFS28690, AAZ07706
	ARBRI-6_T7_B02			
zinc finger	ARBRI-5_T7_A10,	AAD02556, ABN08073	AU09-E09T7, AU08-B12T7	XP_002265999,
	ARBRI-7_T7_H04			XP_002336150
serine protease	ARBRI-C113	CAA07250^*^	AU11_H05T7	CAA07250^*^
beta-galactosidase	ARBRI-C68,	ADV41669, ABN08770	AU12_A03T7	BAH03319
	ARBRI-4_T7_B06			
ubiquitin-like protein	ARBRI-4_T7_G02	XP_002866079	AU12_C05T7	AAZ82816
lipoxygenase	ARBRI-2_T7_F05	ACG56281	AU13_A07T7	ACD43484
nac domain ipr003441	ARBRI-1_T7_D12	XP_002512632	AU13_D11T7, AU04-B01T7	XP_002284654,
				AAM34770
serine hydroxymethyltransferase	ARBRI-C184	CBI17302	AU13_E01T7	ABS72016
glutamine synthetase	ARBRI-C29	AAK08103	AU14_E11T7	BAD94507
60s acidic ribosomal protein p0	ARBRI-C109	XP_002268645	AU-C208	ABB29933
superoxide dismutase	ARBRI-C52,	ADX36104, CAE54085	AU-C109	AAO16563
	ARBRI-7_T7_A02			
cyclophilin	ARBRI-C70	ABS30424^*^	AU04-A01T7	ABS30424^*^
ring finger and chy zinc finger domain-containing protein	ARBRI-C157	AAD02556	AU01-B06T7	XP_002268193
f-box family protein	ARBRI-2_T7_H04	XP_002307387	AU01-G09T7	AAZ81591
glutathione s-transferase	ARBRI-3_T7_D07,	XP_002328824, ADB85090	AU02-E11T7	P46423
	ARBRI-6_T7_B12			
cysteine proteinase precursor	ARBRI-C1	BAF46302	AU01-C03T7	ABK93575
phospholipase d	ARBRI-3_T7_E12	ACG63795	AU07-D08T7	AFK36876
ef hand family protein	ARBRI-8_T7_D07	XP_002319225^*^	AU01-G11T7	XP_002319225^*^
calmodulin	ARBRI-C191	NP_001131288	AU-C96	AAD10247
14-3-3 protein	ARBRI-1_T7_H06	AAY67798	AU11-F06T7	ADK93080

Sequence names, putative protein functions and accession numbers are indicated.

* *ESTs identified with the same accession number in two different libraries (from roots and aerial tissues)*.

### Identification of defense responses induced in aerial tissues during olive root-PICF7 interaction.

**Figure 2:**
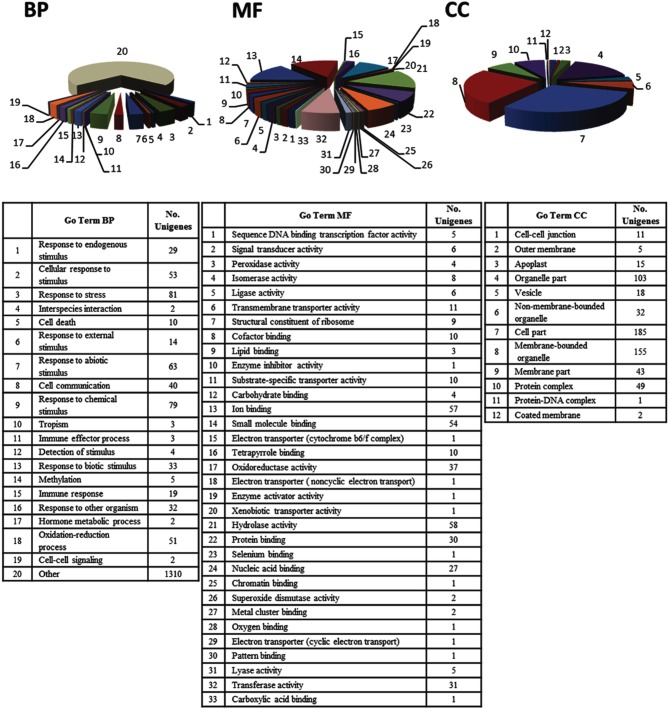
**“Level 3” Gene Ontology (GO) terms distribution of 376 unigenes induced in olive (*Olea europaea* L.) aerial tissues colonized by *Pseudomonas fluorescens* PICF7**. Expressed Sequences Tags (ESTs) were categorized using the “Blast2GO” software according to the terms of the three main GO vocabularies: (BP) “biological processes,” (MF) “molecular functions,” and (CC) “cellular components.” The category “Other” in the main GO vocabulary term “biological processes” clusters 1310 of the transcripts analyzed.

Analysis of the EST set by the Blast2GO software enabled annotation of expressed sequences according to the terms of the three main GO vocabularies, i.e., “biological process” (BP), “molecular function” (MF), and “cellular component” (CC). GO annotation was only feasible for 67.8% of the sequences, i.e., 121 ESTs (106 assigned to “unknown” category and 15 assigned to “predicted” category) were automatically excluded from this functional classification by the program. Since a number of transcripts were identified by different GO terms, the mapped ESTs distribution for BP, MF and CC main categories shown in Figure 2 resulted in more than 376 sequences. The distribution of assignments into the GO categories “level 3” was 208 (BP), 192 (MF), and 150 (CC). Regarding to BP main GO vocabulary, transcripts representing GO terms categories non-related to plant defense processes (e.g., macromolecule metabolic process, developmental maturation, pigment accumulation, chromosome segregation and microtubule-based process), were grouped as “other” (Figure 2, BP).

**Figure 3:**
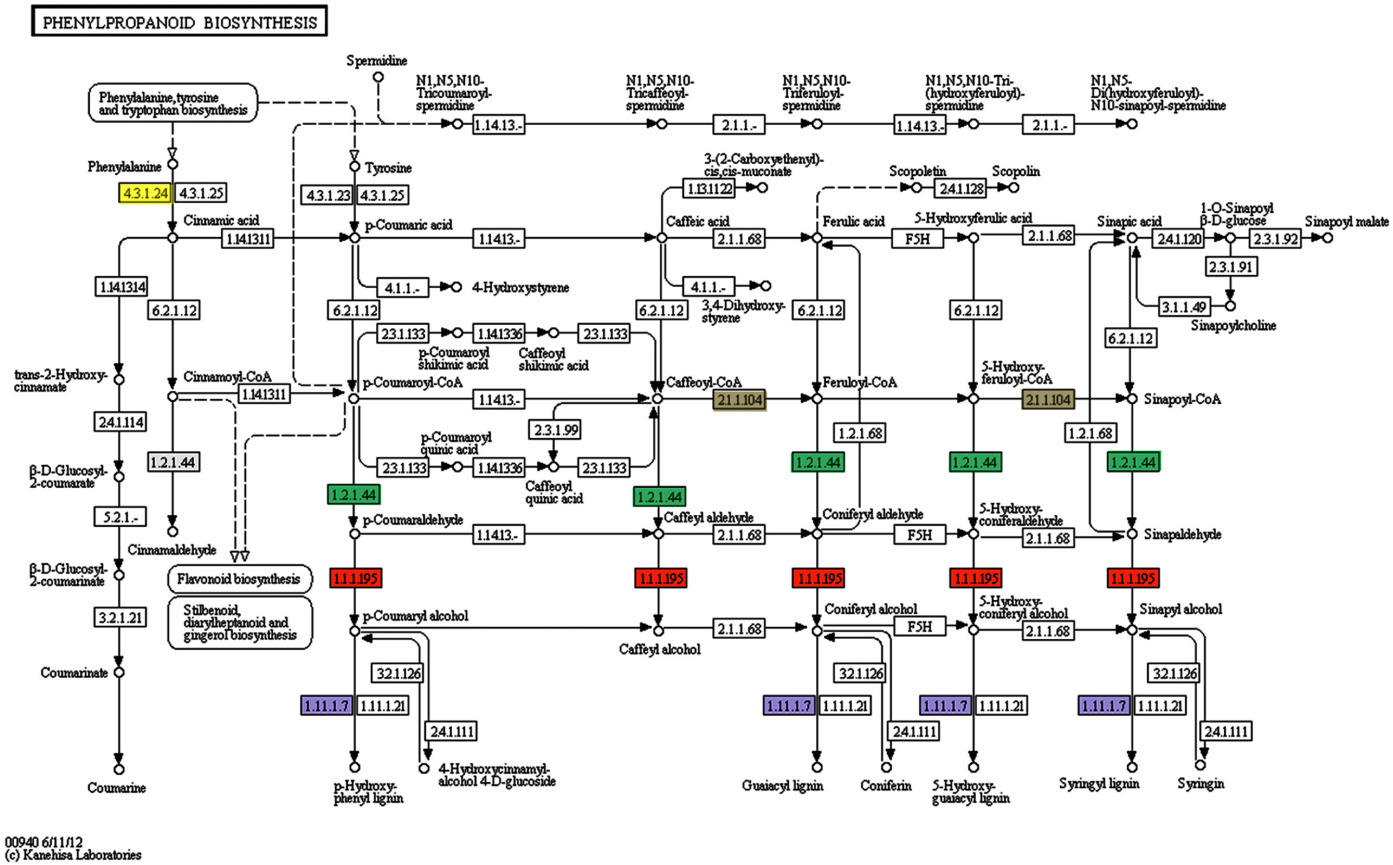
**Patway map from KEGG for the phenylpropanoid biosynthesis pathway**. Several genes involved in this pathway have been found to be induced in olive roots (Schilirò et al., 2012) and/or aerial tissues (this study) upon *Pseudomonas fluorescens* PICF7 root inoculation. The enzymes reductase (1.2.1.44) (green rectangles) induced in aerial tissues, the cinnamyl-alcohol deydrogenase (1.1.1.95) (orange rectangles), the lactoperoxidase (1.11.1.7) (violet rectangles), the phenylalanine ammonia-lyase (4.3.1.5)^*^ induced in roots and aerial tissues, and the caffeoyl-CoA Omethyltransferase (2.1.1.104) (gray rectangles) induced in roots, are mapped. ^*^Phenylalanine ammonia-lyase (EC4.3.1.5) is now divided into three different enzymes: EC4.3.1.23 (tyrosine ammonia-lyase), EC4.3.1.24 (phenylalanine ammonia-lyase), and EC4.3.1.25 (phenylalanine/tyrosine ammonia-lyase) according to IUBMB Enzyme Nomenclature (http://www.chem.qmul.ac.uk/iubmb/enzyme/EC4/3/1/5.html). Yellow rectangle is phenylalanine ammonia-lyase.

Concerning to plant defense-related categories, ESTs found to be induced in above-ground organs upon *P. fluorescens* PICF7 olive root colonization, were assigned to processes such as “response to stress” (81 unigenes), “response to chemical stimulus” (79 unigenes), “response to abiotic stimulus” (63 unigenes), “oxidation-reduction process” (51 unigenes), “response to biotic stimulus” (33 unigenes), or “response to other organism” (32 unigenes). GO terms included CATs [AU03-B01], proteins involved in the phenylpropanoid pathway (PAL [AU03-E02], reductase [AU01-H12], lactoperoxidase [AU02-F09; AU-C159], dehydrogenase [AU08-A06]) (Figure 3), ET biosynthesis (ACO [AU12-D10]) or terpenoids biosynthesis (reductoisomerase [AU-C168]), ACL (AU04-G10), linolenic acid metabolism (monoxygenase [AU05-F07], and LOXs [AU13-A01; AU13-A07]). In addition, we identified transcripts belonging to different classes of PR proteins such as thaumatin-like protein (PR-5) (AU04-G06) or PR protein STH2 (PR-10) (AU05-F04).

For the main GO vocabulary term MF 33 different categories could be identified for “level 3.” The four main categories were “hydrolase activity” (58 unigenes, i.e., epoxide hydrolase 2-like [AU02-A10], raffinose synthase [AU03-E11]), tubby-like f-box protein 8-like [AU09-B03] or alpha beta hydrolase domain [AU-C51]) “ion binding” and “small molecule binding” (with 57 unigenes, i.e., LOXs [AU13-A01; AU13-A07], ACO [AU12-D10], nucleoside diphosphate kinase [AU12-B11] or serine hydroxymethyltransferase [AU13-E01]) and 54 unigenes, i.e., cysperoxiredoxin [AU02-F09], serine hydroxymethyltransferase [AU13-E01], or nucleoside diphosphate kinase [AU12-B11]) and “oxidoreductase activity” (37 unigenes, i.e., phosphoglycerate mutase [AU05-D07], ACO [AU12-D10], histidinol dehydrogenase [AU01-F08] or cytochrome p450 [AU08-E04]) (Figure 2, MF).

Finally, most of the unigenes identified for the main GO term vocabulary CC were assigned to: “cell part” (185 unigenes, i.e., protein ET insensitive [AU08-H12], phosphoribulokinase precursor [AU07-H02], CAT [AU03-B01], thiredoxin-like protein [AU02-C01], ribosomal protein s1 [AU04-C09]), “membrane-bounded organelle” (155 unigenes, i.e., loxc homolog [AU13-A01], cytochrome b6 [AU-C41], photosystem i reaction center subunit n [AU14-C11], nucleoporin autopeptidase [AU12-C08]), and “organelle part” (103 unigenes, i.e., auxin response factor 9 [AU01-H05], transcription factor bHLH 110-like [AU02-B01]) (Figure 2, CC).

### Time-course of expression and validation analysis of selected defense response olive genes induced by strain PICF7

A qRT-PCR time-course study was carried out to validate gene expression of five selected genes identified as induced in olive aerial tissues and present in the generated EST library: *ACO* [AU12-D10], *PAL* [AU03-E02], *CAT* [AU03-B01], *LOX-2* [AU13-A07], and *ACL* [AU04-G10]. Moreover, we aimed to analyze the gene expression pattern along time: i.e., at 1, 3, 7, and 15 DAI of strain PICF7 in olive roots.

**Figure 4:**
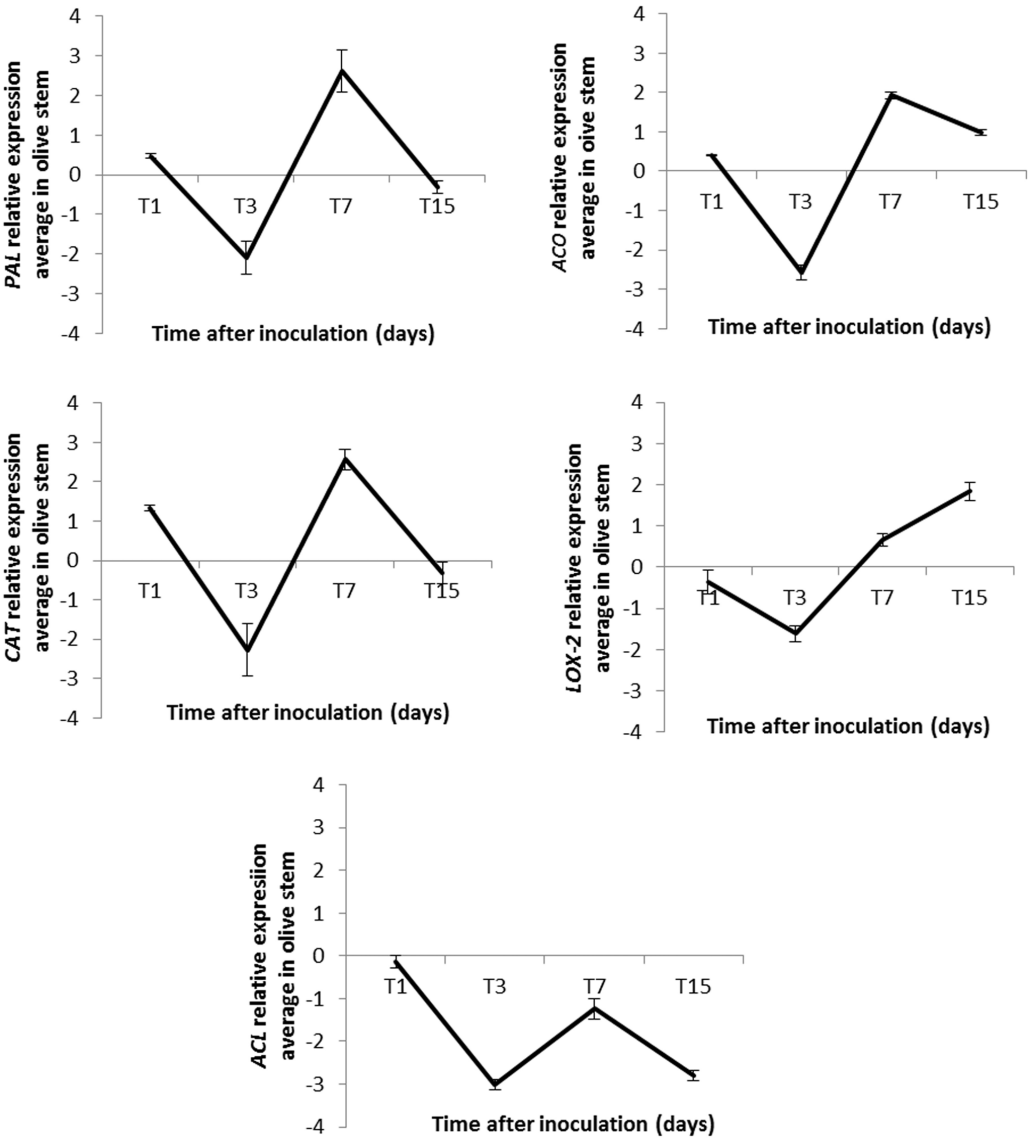
**Relative expression (RE) average of five genes identified as up-regulated in stems of inoculated “Arbequina” olive plants at different time points after *Pseudomonas fluorescens* PICF7 inoculation in roots**. *PAL*, phenylananine ammonia-lyase; *ACO*, 1-aminocyclopropane-1-carboxylate oxidase; *CAT*, catalase; *LOX-2*, lipoxygenase 2; *ACL*, acetone cyanohydrin lyase. Error bars represent the STD from at least two independent experiments. RE values (log_2_ fold-change values) were calculated according to the 2^−ΔΔ*Ct*^ method (Livak and Schmittgen, 2001).

qRT-PCR experiments validated the results from the generated SSH cDNA library for four (*ACO, PAL, CAT*, and *LOX-2*) of the five selected genes, although gene expression patterns varied along time (Figure 4). Linear equations, correlation coefficients (*R*^2^) and PCR efficiencies for each case are shown in Table 3. The relative fold changes were assigned to three categories of up-regulation: high (>+2), medium (>+1.0 to ≤+2.0) and low (≥–1.0 to ≤+1.0), according to Kim et al. (2008).

Overall, results showed a decrease in the transcription level of the analyzed genes 3 days after PICF7 inoculation, and an increase 7 days after introducing the BCA in the olive root system (Figure 4). In three out of the five studied genes we observed up-regulation with maximal expression at 7 DAI, two of them (*PAL* and *CAT*) up-regulated at a high-level and one (*ACO*) at a medium-level (estimated fold change of PAL [+2.62], ACO [+1.93], and *CAT* [+2.57] log units compared with the control, non-bacterized samples) (Figure 4). The increase of *LOX-2* gene expression level was confirmed at 7 and 15 DAI ([+0.66] and [+1.84], respectively; Figure 4). Finally, up-regulation of the putative *ACL* gene could not be corroborated by qRT-PCR analysis, since this transcript showed a down regulation in all measured times (−0.14, −3.02, −1.24, and −2.80) (Figure 4).

## Discussion

Plants can deploy a range of chemical and physical defense barriers to minimize or overcome damages produced at the primary site where any given biotic and/or abiotic stress is acting. Because defense is metabolically costly, plants have evolved inducible defensive mechanisms that can be activated or amplified in response to stress (Walters and Heil, 2007). Specific signals can “prime” plant tissues, preparing them for an augmented response to confront future damage without direct activation of costly defense mechanisms (Conrath, 2009; Jung et al., 2009). In addition, plants have developed countermeasures aimed to halt the dispersal of deleterious (micro)organisms therefore preventing further damage at distant parts (Gaupels and Vlot, 2012). On the other hand, beneficial soil-borne microorganisms, such as mycorrhizal fungi and PGPR, can induce systemic plant immunity (Van Loon et al., 1998; Pozo and Azcon-Aguilar, 2007; Zamioudis and Pieterse, 2012). Plants thus must deal with a broad range of microorganisms (deleterious and beneficial) that interact with them at any time thereby influencing their defense response (Van der Putten et al., 2001; Stout et al., 2006; Poelman et al., 2008). Hence, plants need efficient regulatory mechanisms to effectively adapt to different trophic interactions. In this scenario, crosstalk among hormone-signaling pathways provides plants a powerful regulatory potential to defend themselves against a range of invaders (Reymond and Farmer, 1998; Kunkel and Brooks, 2002; Bostock, 2005; Pieterse and Dicke, 2007; Pieterse et al., 2009).

A functional genomics study was conducted to shed light on potential systemic genetic responses taking place in aerial tissues during the interaction between the biocontrol endophyte *P. fluorescens* PICF7 and olive root tissues. Computational analysis revealed that 376 transcripts were induced upon PICF7 treatment, and that many of them were potentially involved in plant defense and response to different kind of stresses. This demonstrates that PICF7 is able to mount systemic defense responses at distant tissues from its natural colonization niche (roots). Strain PICF7 was previously shown to effectively colonized inner root tissues with no evidence of translocation to above-ground organs (Prieto and Mercado-Blanco, 2008; Prieto et al., 2009, 2011; Maldonado-González et al., 2013). Therefore, the effective biocontrol displayed by PICF7 against VWO (Mercado-Blanco et al., 2004; Prieto et al., 2009), which can be explained by the induction of defense response at the root level (Schilirò et al., 2012), seems also to operate systemically. Furthermore, some of the genes involved in defense response here reported were also up-regulated in root tissues (Schilirò et al., 2012). This suggests that strain PICF7 could potentially be an effective BCA against olive pathogens other that *V. dahliae* through an ISR mechanism. However, root inoculation of *in vitro*-propagated olive plants with PICF7 did not hinder olive knot (caused by *Pseudomonas savastanoi* pv. savastanoi) development in stems (Maldonado-González et al., 2013). Thus, while PICF7 is able to trigger a range of systemic defense responses, they seem to be ineffective at least against this phytopathogenic bacterium.

Our results indicated that, among others, genes involved in plant hormones and phenylpropanoids biosynthesis (i.e., *PAL, ACL, LOX-2*, etc.), oxidative stress (*CAT*), and Ca^2+^ metabolism (CaM, glutamate descarboxylase, or an ef hand family protein) implicated in systemic defensive responses are induced in aerial olive tissues during the interaction of PICF7 with olive roots. Besides, several transcription factors related to plant defense were found as up-regulated, i.e., JERF [implicated in regulation of plant stress and response to fungal disease attack (Zhang et al., 2004)], WRKY [global regulators of host responses following challenge by phytopathogenic organisms (Pandey and Somssich, 2009)], and bHLH [involved in response to pathogen (Van Verk et al., 2009)]. Among the ample set of transcripts found to be up-regulated in the generated SSH library, we selected for validation purpose and to assess expression pattern along time five genes related with plant defense responses. ACC oxidase (ACO) catalyzes the final step in the biosynthesis of ET, known as the “stress hormone” which is regulated by diverse environmental factors, including (a)biotic stresses (Wang et al., 2002). For instance, *ACO* mRNA levels, as well as ACO activity, can be elevated under phytopathogen attacks (Díaz et al., 2002; Iwai et al., 2006). Moreover, it has been proved that *ACO* gene silencing can affect the susceptible host response to pathogen (Shan and Goodwin, 2006). Our results indicate that a putative olive *ACO* [AU12-D10] was moderately up-regulated in aerial tissues upon root colonization by PICF7, suggesting an ET-mediated systemic defense response triggered when a beneficial bacterial endophyte is introduced in the root system.

CATs are involved in decomposition of H_2_O_2_ into H_2_O and O_2_. A close interaction has been reported between intracellular H_2_O_2_ and cytosolic Ca^2+^ concentration in response to both biotic and abiotic stresses (Rudd and Franklin-Tong, 2001; Yang and Poovaiah, 2002; White and Broadley, 2003). These studies indicate that an increase in cytosolic Ca^2+^ boosts the generation of H_2_O_2_. The Ca^2+^-binding protein CaM activates some plant CATs in the presence of Ca^2+^. Yang and Poovaiah (2002) proposed that increased cytosolic Ca^2+^ has a dual role. For positive regulation, extracellular signals trigger an influx of Ca^2+^ which increases H_2_O_2_ levels; for negative regulation, Ca^2+^ binds to CaM, and this complex stimulates CAT activity leading to the rapid degradation of H_2_O_2_. The increase in H_2_O_2_ can enhance the Ca^2+^ influx by activating the Ca^2+^ channel (Pei et al., 2000). Our results showed that a putative olive *CAT* [AU03-B01] was highly induced (+2.57) at 7 DAI. Besides, a *CAT* gene (same accession number) was also up-regulated in olive roots (Schilirò et al., 2012). Interestingly, CaM and other Ca^2+^-related proteins have been found to be induced in olive aerial tissues as well (i.e., CaM [AU-C96], ef hand family protein [AU01-G11] and calreticulin [AU02-C02]). This suggests that the complex Ca^2+^/CaM can decrease H_2_O_2_ levels in plants by stimulating CATs activities and hence their possible role in plant defense responses (Yang and Poovaiah, 2002). It might well be that olive plants first react (even systemically) to the invasion of PICF7 by the induction of *CAT*. However, this response seems to be attenuated at later times (15 DAI). It is tempting to speculate that the decrease in *CAT* expression (also observed for other validated genes) could be due to the fact that this endophytic bacterium is eventually recognized by the host plant as a non-hostile microorganism, enabling its endurance inside the plant. Alternatively, strain PICF7 might counteract this defense response by deploying an as yet unidentified mechanism(s).

The activation of systemic resistance by non-pathogenic rhizobacteria has been also associated with the induction of LOX activity in plants such as bean (*Phaseolus vulgaris* L.) and tomato (*Solanum lycopersicum* Mill) (Blée, 2002; Silva et al., 2004; Ongena et al., 2007). Plant LOX catalyzes incorporation of O_2_ in polyunsaturated fatty acids to yield the corresponding fatty acid hydroperoxides. These are related to substrates for other enzymes leading to the production of JAs involved in signaling events and regulation of plant defense gene expression (Feussner and Wasternack, 2002; Shah, 2005). A putative olive *LOX-2* [AU13-A07], involved in linolenic acid metabolism (Siedow, 1991), was found to be up-regulated at latter times after bacterization (15 DAI, +1.84). Another *LOX*, implicated in JA biosynthesis, was found to be up-regulated in olive roots (Schilirò et al., 2012). This fact further supports the possible role of PICF7 in triggering olive defense response not only in roots but also in above-ground tissues.

A putative olive *ACL* [AU04-G10] was found to be induced in aerial tissues by the SSH approach implemented in this study. Up regulation of *ACL* was previously reported in root tissues upon strain PICF7 colonization (Schilirò et al., 2012). Therefore, ACL activity seems to be induced at both local and systemic levels in olive plants when interacting with PICF7. This enzyme is involved in the catabolism of cyanogenic glycosides (Trummler and Wajant, 1997) which play pivotal roles in the organization of chemical defense systems in plants against pathogens and herbivores and in plant-insect interactions (Ganjewala et al., 2010). However, induction of *ACL* [AU04-G10] was not validated at the time points checked by qRT-PCR experiments. A possible explanation for this outcome is that *ACL* is induced in a very transient way and/or at very specific time points other than those assessed in this study.

The expression pattern of a gene coding for a putative olive *PAL* [AU03-E02] was also evaluated. *PAL* genes can be induced by wounding, low temperature, pathogen attack, and other stress conditions (Collinge and Slusarenko, 1987; Wu and Lin, 2002), and activation of the phenylpropanoids pathway in plants is linked to diverse stress situations (Gómez-Vásquez et al., 2004). The induction of the *PAL* gene [both in roots (Schilirò et al., 2012) and aerial tissues(this study)] in olive upon PICF7 treatment suggests that this defense response pathway, as others analyzed in this study, is a consequence of PICF7 colonization being recognized as a stressful situation by the host plant, at least transiently (maximum relative expression at 7 DAI, +2.62). On the other hand, PAL activation can play a role in biocontrol activity displayed by strain PICF7, as reported for other plant-endophyte interactions (Benhamou et al., 1996a,b; Ramamoorthy et al., 2002).

Our previous results have shown that root colonization by PICF7 induced a broad range of defense response genes in root tissues (Schilirò et al., 2012). This present study demonstrates that many of these responses are also systemically up-regulated in aerial tissues: genes involved in plant hormones and phenylpropanoids biosynthesis, PR proteins, and several transcription factors involved in systemic defensive responses. In fact, 26 up-regulated transcripts detected in aerial tissues were annotated with the same putative function than that of genes induced in olive roots upon PICF7 colonization (i.e., PAL, ACL, CAT, LOX, 14-3-3 protein, CaM, thaumatin-like protein, etc.) (Table 3). Our study therefore provides a database of differentially-expressed transcripts deserving future research. It constitutes an excellent starting point for in-depth genetic analysis to further characterize the interaction between plants and beneficial bacterial endophytes. Some genes could possibly constitute specific markers distinguishing this type of plant-microbe interaction from other trophic scenarios such as plant-pathogen and/or plant-symbiont interactions. Alternatively, commonalities among these interactions could also be uncovered. For instance, a putative gene coding for a 14-3-3 protein was found to be up regulated in both roots (Schilirò et al., 2012) and aerial tissues (this study) upon PICF7 root colonization (Table 3). Manosalva et al. (2011) have reported that a rice 14-3-3 protein (GF14e) negatively affected the induction of plant defense response genes, cell death and disease resistance in this host. It would be interesting to investigate whether the induction of this gene in olive might contribute to facilitate endophytic colonization by PICF7 because of defense responses mediated by SA or reactive oxygen species (as cell death or SAR) are being attenuated by this protein.

Interestingly enough, all transcripts evaluated by qRT-PCR showed a decrease in their relative expression at 3 DAI and an increase at 7 DAI. We do not have a clear answer for this fluctuation. Without ruling out other explanations, a possible reason could be that plants were not exposed to a complete daylight period along the first 5 days of the bioassay. Indeed, in order to protect plants from excessive light stress after uprooting, cleaning and inoculation procedures, the day–night cycle was progressively applied until reaching the complete 14-h photoperiod (see Materials and Methods). Little is known about how external abiotic factors, for instance light exposure and intensity, can influence the ability of plants to defend from biotic stresses. A few reports point to a light dependency of distinct defense responses in different systems (Graham and Graham, 1996; Asai et al., 2000; Brodersen et al., 2002). Zeier et al. (2004) reported that whereas *PAL* transcripts accumulated in Arabidopsis leaves 2–6 h post infection with *P. syringae* pv. maculicola (avrRpm1) under medium or high light conditions, they failed to do so at dark. Therefore, light (duration and intensity) could play an important regulatory role influencing disease resistance responses. On the other hand, the observed overall increase in transcripts level at 7 DAI could be related to the moment in which plant defense responses here analyzed reached their maximum expression level in aerial tissues in response to root bacterial colonization. The subsequent decrease observed at 15 DAI could indicate that the presence of this endophytic bacterium in roots is somehow recognized as “non-hostile.” Therefore, initially-induced defense responses are eventually modulated/attenuated allowing the establishment of this beneficial association, as reported elsewhere (Plucani do Amaral et al., 2014).

In summary, we have demonstrated that PICF7 is able to activate an array of defense pathways not only in olive root tissues (Schilirò et al., 2012) but also at distant parts of the plant. This is one of the first studies demonstrating that root colonization by a beneficial endophyte triggers systemic responses. Moreover, this has been accomplished in a woody plant such as olive and using non-gnotobiotic conditions. On the one hand, our functional genomics approach can shed light on how the plant broadly and systemically respond to a specific interaction (i.e., colonization by a beneficial endophytic bacterium), a trophic scenario poorly investigated within plant-microbe interactions studies. Olive plants seem to react to a “non-hostile” colonization by deploying several defense responses that eventually must be modulated or attenuated to ensure penetration, colonization, and survival of PICF7 cells inside root tissues. Alternatively, PICF7 can also be able to counteract these responses by specific, unidentified traits enabling this bacterium to be recognized as a harmless invader. On the other hand, the genetic responses triggered by PICF7, even at distant tissues, may explain its biocontrol activity. How effective and durable are these responses and what are the bacterial traits involved in the endophytic lifestyle of PICF7 are matters of ongoing studies.

### Conflict of interest statement

The authors declare that the research was conducted in the absence of any commercial or financial relationships that could be construed as a potential conflict of interest.
