# Supplementary material for: The biocontrol endophytic bacterium Pseudomonas fluorescens PICF7 induces systemic defense responses in aerial tissues upon colonization of olive roots
Source: Front Microbiol. 2014 Sep 5;5:427. doi: 10.3389/fmicb.2014.00427 (PMC4155815; doi:10.3389/fmicb.2014.00427)
Supplement: Supplementary file 1 [file DataSheet1.DOCX]

| **Table S1**. List of EST sequences induced in aerial olive tissues (cv. Arbequina) by the biocontrol, endophytic strain *Pseudomonas fluorescens* PICF7. The EST Sequence name refers to the codes assigned within the cDNA library. AU means Arbequina aerial tissues induced gene and AU-C indicates Arbequina aerial tissues inducted as part of a contig. T7 refers to the forward T7 universal primers used for sequencing. Homologous genes were identified in the GenBank protein database (non-redundant) by running the Blastx algorithm set to 1.0 E-3 (in Blast2GO v. 2.7.0). ESTs homologous to protein with unknown function are not included in this table. EST sequence name, putative protein function, organism, accession number, and related E-value are shown | | | | |
| --- | --- | --- | --- | --- |
| **EST Sequence name** | **Putative protein function** | **Organism** | **Accession Number** | **E-Value** |
| AU01-A06T7 | uroporphyrinogen decarboxylase | *Vitis vinifera* | XP_002274385 | 4,10E-76 |
| AU01-B01T7 | s-adenosyl-l-methionine-dependent methyltransferases superfamily | *Olea europaea* | AFS28695 | 4,76E-48 |
| AU01-B03T7 | superoxide dismutase | *Haberlea rhodopensis* | ADX36104 | 3,86E-54 |
| AU01-B06T7 | ring finger and chy zinc finger domain-containing protein | *Vitis vinifera* | XP_002268193 | 1,91E-31 |
| AU01-B12T7 | plastid-lipid-associated protein | *Populus trichocarpa* | XP_002333019 | 2,87E-39 |
| AU01-C02T7 | cysteine proteinase aalp | *Platycodon grandiflorus* | BAF81994 | 2,79E-68 |
| AU01-C03T7 | papain family cysteine protease | *Populus trichocarpa* | ABK93575 | 1,35E-80 |
| AU01-C08T7 | glutamine synthetase | *Oryza sativa Indica* Group | ABR25621 | 5,14E-33 |
| AU01-D01T7 | at3g42670-like partial | *Vitis vinifera* | XP_002273775 | 2,02E-65 |
| AU01-D04T7 | beta-glucosidase 13-like | *Olea europaea* subsp*. europaea* | AAL93619 | 4,93E-61 |
| AU01-D09T7 | extra-large g- | *Cucumis melo* subsp*. melo* | ADN34175 | 9,11E-52 |
| AU01-E02T7 | clathrin assembly protein at2g01600-like | *Vitis vinífera* | XP_002284692 | 7,90E-64 |
| AU01-E05T7 | polygalacturonase inhibitor protein | *Antirrhinum majus* subsp*. cirrhigerum* | AAL08699 | 1,48E-22 |
| AU01-E07T7 | leucine-rich repeat receptor-like serine threonine-protein kinase bam1-like | *Medicago truncatula* | XP_003608587 | 2,07E-53 |
| AU01-E09T7 | chlorophyll a-b binding protein | *Petunia x hybrida* | P13869 | 8,18E-82 |
| AU01-E11T7 | at1g15230 f9l1_18 | *Populus trichocarpa* | XP_002304198 | 1,16E-18 |
| AU01-F07T7 | protein | *Glycine max* | XP_003547858 | 1,32E-17 |
| AU01-F08T7 | histidinol dehydrogenase | *Humulus lupulus* | ABS17594 | 6,26E-11 |
| AU01-G08T7 | atp-dependent zinc metalloprotease ftsh chloroplastic-like | *Vitis vinífera* | CBI22027 | 9,76E-48 |
| AU01-G09T7 | f-box protein pp2-b10-like | *Nicotiana tabacum* | AAZ81591 | 3,31E-18 |
| AU01-G11T7 | ef hand family protein | *Populus trichocarpa* | XP_002319225 | 1,63E-39 |
| AU01-H04T7 | protein | *Populus trichocarpa* | XP_002332166 | 2,89E-33 |
| AU01-H05T7 | auxin response factor 9 | *Vitis vinífera* | CBI34510 | 2,85E-42 |
| AU01-H12T7 | glycine-rich rna-binding protein | *Medicago truncatula* | XP_003606921 | 4,28E-39 |
| AU02-A03T7 | salt tolerance-related protein | *Olea europaea* | ABS72020 | 3,53E-60 |
| AU02-A05T7 | beta-glucosidase d4 | *Olea europaea* | ACD43481 | 1,23E-54 |
| AU02-A06T7 | protein phosphatase 2c 16 | *Solanum lycopersicum* | NP_001234686 | 1,81E-22 |
| AU02-A07T7 | photosystem ii reaction center w chloroplast | *Populus trichocarpa* x *Populus deltoides* | ABK96462 | 7,64E-15 |
| AU02-A10T7 | epoxide hydrolase 2-like | *Citrus jambhiri* | BAD13534 | 6,37E-54 |
| AU02-B01T7 | transcription factor bhlh110-like | *Vitis vinifera* | XP_002281118 | 1,25E-30 |
| AU02-B10T7 | chlorophyll a b-binding protein cab- partial | *Vitis vinifera* | CBI30836 | 3,81E-24 |
| AU02-C01T7 | thioredoxin-like protein | *Populus trichocarpa* | XP_002322732 | 4,27E-69 |
| AU02-C02T7 | calreticulin | *Pinus taeda* | AAG01147 | 7,71E-42 |
| AU02-C04T7 | Chain A family protein | *Populus trichocarpa* | XP_002335027 | 2,89E-15 |
| AU02-C06T7 | mannose-6-phosphate isomerase | *Vitis vinifera* | CBI33642 | 1,50E-30 |
| AU02-C12T7 | ribulose bisphosphate carboxylase oxygenase activase chloroplastic-like | *Olea europaea* | ABS72022 | 9,81E-23 |
| AU02-D01T7 | protein | *Populus trichocarpa* | XP_002307245 | 7,26E-31 |
| AU02-D05T7 | adp-glucose pyrophosphorylase large subunit | *Ipomoea batatas* | CAB51610 | 3,43E-137 |
| AU02-D06T7 | ubiquitin protein 2 | *Platanus x acerifolia* | CAL07979 | 8,78E-19 |
| AU02-E06T7 | chloroplast ribulose- -bisphosphate carboxylase oxygenase small subunit | *Olea europaea* | ABS71998 | 3,51E-10 |
| AU02-E11T7 | glutathione s-transferase | *Hyoscyamus muticus* | P46423 | 1,24E-27 |
| AU02-F03T7 | eto1-like protein 1-like | *Zea mays* | NP_001182847 | 9,91E-52 |
| AU02-F05T7 | gdp-d-mannose-3 -epimerase | *Lotus japonicus* | AFK43031 | 5,95E-35 |
| AU02-F06T7 | nuclear export mediator factor nemf-like | *Vitis vinifera* | XP_002273922 | 1,97E-16 |
| AU02-F09T7 | 2-cys peroxiredoxin | *Ricinus communis* | XP_002530152 | 1,74E-19 |
| AU02-F12T7 | blight-associated protein p12 | *Alnus glutinosa* | ADG29118 | 5,02E-03 |
| AU02-G03T7 | chlorophyll a-b binding protein 4 precursor homolog | *Vitis vinifera* | XP_002284856 | 6,63E-58 |
| AU02-G09T7 | photosystem ii oxygen-evolving complex protein 3 | *Spinacia oleracea* | P12301 | 2,12E-17 |
| AU02-G12T7 | ubiquitin carrier protein e2 | *Ricinus communis* | XP_002519212 | 2,36E-09 |
| AU02-H04T7 | protein | *Populus trichocarpa* | XP_002310966 | 2,50E-16 |
| AU02-H09T7 | cytochrome p450 | *Sesamum indicum* | AAZ07706 | 2,17E-89 |
| AU02-H12T7 | gdsl esterase lipase | *Jacaranda mimosifolia* | ABY59947 | 1,73E-60 |
| AU03-A02T7 | elongation factor 1-beta | *Solanum tuberosum* | ABA40427 | 6,09E-46 |
| AU03-A04T7 | auxin-regulated protein | *Eucommia ulmoides* | ABW24025 | 7,97E-13 |
| AU03-A07T7 | predicted protein | *Populus trichocarpa* | XP_002309595 | 6,65E-14 |
| AU03-B01T7 | catalase | *Tectona grandis* | CBA13361 | 8,08E-56 |
| AU03-B03T7 | ubiquitin-associated ts-n domain-containing protein | *Vitis vinifera* | XP_002273725 | 1,88E-24 |
| AU03-B04T7 | jaz1-like protein | *Ipomoea batatas* | ABP35523 | 2,70E-17 |
| AU03-B11T7 | polyubiquitin-like protein | *Citrus maxima* | ADC29516 | 3,45E-54 |
| AU03-B12T7 | leucine-rich repeat receptor-like serine threonine tyrosine-protein kinase sobir1-like | *Nicotiana tabacum* | BAC07504 | 3,51E-64 |
| AU03-D06T7 | hypothetical protein ARALYDRAFT_902175 | *Arabidopsis lyrata* subsp*. Lyrata* | XP_002881174 | 3,43E-07 |
| AU03-D02T7 | 21 kda protein | *Vitis vinifera* | XP_002264028 | 3,87E-18 |
| AU03-E02T7 | phenylalanine ammonia-lyase | *Scutellaria baicalensis* | ABS58596 | 2,43E-99 |
| AU03-E04T7 | sec13-related protein | *Vitis vinifera* | XP_002265971 | 1,68E-26 |
| AU03-E07T7 | mitochondrial substrate carrier family protein b-like | *Vitis vinifera* | XP_002278430 | 4,06E-52 |
| AU03-E11T7 | raffinose synthase | *Boea hygrometrica* | AEP68101 | 3,01E-16 |
| AU03-F03T7 | photosystem ii oxygen-evolving complex protein 3-like | *Bruguiera gymnorhiza* | BAA96362 | 1,56E-25 |
| AU03-F06T7 | transcription factor une12 | *Vitis vinifera* | XP_002284047 | 7,99E-52 |
| AU03-F12T7 | psal protein | *Olea europaea* | ABU39903 | 4,26E-48 |
| AU03-G03T7 | aspartic proteinase nepenthesin-2 | *Lotus japonicus* | AFK48193 | 3,51E-27 |
| AU03-G04T7 | leucine-rich repeat family protein protein kinase family protein | *Ricinus communis* | XP_002515020 | 9,53E-06 |
| AU03-G06T7 | 60s ribosomal protein l27 | *Vitis vinifera* | XP_002277425 | 3,21E-06 |
| AU03-G11T7 | glycine-rich rna-binding protein | *Catharanthus roseus* | AAF31403 | 2,28E-33 |
| AU03-H02T7 | atp synthase cf1 alpha partial | *Coffea buxifolia* | AFI55876 | 5,67E-123 |
| AU03-H10T7 | protein chloroplastic-like isoform x2 | *Vitis vinifera* | XP_003633960 | 2,07E-57 |
| AU04-A01T7 | cyclophilin | *Nicotiana tabacum* | ABS30424 | 2,05E-70 |
| AU04-A03T7 | glutamate decarboxylase | *Glycine max* | XP_003538378 | 4,67E-15 |
| AU04-A05T7 | 60s ribosomal protein l21 | *Paeonia suffruticosa* | ABQ65185 | 1,03E-11 |
| AU04-B01T7 | nac domain ipr003441 | *Petunia x hybrida* | AAM34770 | 1,40E-31 |
| AU04-B04T7 | multidrug pheromone mdr abc transporter family | *Vitis vinifera* | XP_002279471 | 3,13E-33 |
| AU04-B08T7 | multiprotein bridging factor 1 | *Nicotiana tabacum* | BAB88859 | 4,10E-41 |
| AU04-B12T7 | membrane | *Arabidopsis thaliana* | NP_568009 | 1,37E-56 |
| AU04-C08T7 | cellulose synthase-like | *Nicotiana tabacum* | AAZ79231 | 2,32E-04 |
| AU04-C09T7 | ribosomal protein s1 | *Spinacia oleracea* | P29344 | 2,29E-23 |
| AU04-D06T7 | hypothetical protein VITISV_039443 | *Vitis vinífera* | CAN80273 | 7,35E-16 |
| AU04-D12T7 | glucose-6-phosphate isomerase | *Vitis vinífera* | CBI19242 | 5,53E-96 |
| AU04-E03T7 | adp-glucose pyrophosphorylase small subunit | *Zea mays* | DAA49239 | 8,71E-11 |
| AU04-E08T7 | nadh dehydrogenase | *Solanum tuberosum* | NP_001275317 | 1,69E-25 |
| AU04-E10T7 | jasmonate zim-domain protein | *Nicotiana tabacum* | BAG68656 | 4,67E-12 |
| AU04-E12T7 | poly -binding protein | *Plantago major* | CAJ38367 | 3,05E-26 |
| AU04-F06T7 | rab family gtpase | *Medicago truncatula* | ACJ84148 | 9,11E-26 |
| AU04-F08T7 | sin3 histone deacetylase complex | *Vitis vinifera* | XP_002281791 | 8,85E-82 |
| AU04-F11T7 | phosphoglycerate bisphosphoglycerate mutase family protein | *Nicotiana tabacum* | CCE46056 | 3,28E-33 |
| AU04-F12T7 | glutamyl-trna synthetase | *Medicago truncatula* | XP_003591273 | 4,30E-16 |
| AU04-G06T7 | thaumatin-like protein | *Olea europaea* | ACZ57583 | 1,09E-25 |
| AU04-G10T7 | protein | *Nicotiana tabacum* | Q6RYA0 | 1,64E-25 |
| AU05-A07T7 | btb poz domain-containing protein at5g41330-like | *Vitis vinífera* | XP_003631414 | 1,63E-11 |
| AU05-A08T7 | senescence-associated protein | *Lilium longiflorum* | ABO20848 | 8,82E-38 |
| AU05-A09T7 | gonadotropin beta chain | *Ricinus communis* | XP_002520219 | 5,13E-41 |
| AU05-A12T7 | early-responsive to dehydration 4 | *Davidia involucrata* | AAL47004 | 2,86E-27 |
| AU05-B06T7 | protein | *Ricinus communis* | XP_002533928 | 4,91E-05 |
| AU05-C02T7 | aldehyde dehydrogenase 7b4 | *Sorghum bicolor* | AAB47996 | 3,87E-22 |
| AU05-C06T7 | gdsl esterase lipase | *Arachis hypogaea* | ADY38375 | 8,49E-68 |
| AU05-C07T7 | 60s acidic ribosomal protein p1 | *Physcomitrella patens* subsp*. patens* | XP_001771167 | 3,60E-24 |
| AU05-C10T7 | protein | *Vitis vinífera* | CAN73103 | 2,73E-10 |
| AU05-C12T7 | cysteine proteinase inhibitor | *Knorringia sibirica* | ADD69946 | 1,06E-07 |
| AU05-D07T7 | phosphoglycerate mutase-like protein | *Populus trichocarpa* | XP_002323826 | 4,92E-82 |
| AU05-D12T7 | dentin sialophospho protein | *Vitis vinífera* | CAN75603 | 1,22E-32 |
| AU05-F04T7 | pathogenesis-related protein | *Salvia miltiorrhiza* | ABR10301 | 1,19E-11 |
| AU05-F05T7 | serine threonine-protein phosphatase 6 regulatory subunit 3-like | *Vitis vinífera* | CBI16320 | 3,80E-54 |
| AU05-F07T7 | cytochrome p450 liketbp | *Micromonas pusilla* | XP_003064993 | 4,06E-35 |
| AU05-F08T7 | 1-deoxy-d-xylulose 5-phosphate reductoisomerase | *Olea europaea* | AFS28671 | 3,75E-59 |
| AU05-H03T7 | protein in2-1 homolog b-like | *Vitis vinífera* | CBI32630 | 2,69E-07 |
| AU06-A10T7 | protein tify 10a-like | *Zea mays* | NP_001182812 | 5,38E-06 |
| AU06-B02T7 | cell division control protein | *Nicotiana glutinosa* | ACS28251 | 2,79E-26 |
| AU06-B04T7 | elongation factor g | *Zea mays* | NP_001168155 | 5,01E-78 |
| AU06-B05T7 | amino acid selective channel protein | *Ricinus communis* | XP_002532855 | 1,77E-37 |
| AU06-C08T7 | protein smg7-like | *Vitis vinífera* | CAN78121 | 1,76E-30 |
| AU06-D07T7 | disease resistance response protein 206 | *Vitis vinífera* | CAN76521 | 1,77E-25 |
| AU06-D12T7 | developmentally regulated g-protein 2 | *Oryza sativa Japonica* Group | NP_001055297 | 1,15E-08 |
| AU06-F07T7 | acyl-activating enzyme 17 | *Populus trichocarpa* | XP_002338384 | 1,17E-46 |
| AU06-H04T7 | pinus taeda anonymous locus 2_4079_01 genomic sequence | *Vitis vinífera* | XP_002277128 | 3,55E-27 |
| AU06-H07T7 | catalase | *Prunus persica* | CAB56850 | 5,35E-73 |
| AU07-B01T7 | costars family | *Vitis vinífera* | XP_002270515 | 6,78E-35 |
| AU07-B02T7 | calreticulin-3- partial | *Medicago truncatula* | ACJ85691 | 3,34E-41 |
| AU07-B07T7 | protein transport protein sec61 subunit alpha | *Zea mays* | NP_001136901 | 5,64E-74 |
| AU07-B12T7 | 60s ribosomal protein l37a | *Vitis vinífera* | XP_002282974 | 1,46E-51 |
| AU07-D03T7 | ubiquitin thioesterase otubain-like protein | *Ricinus communis* | XP_002534741 | 2,06E-126 |
| AU07-D08T7 | phospholipase d | *Lotus japonicus* | AFK36876 | 1,85E-105 |
| AU07-E07T7 | translation initiation factor eif-5b | *Pisum sativum* | AAN32916 | 1,76E-72 |
| AU07-F01T7 | expansin-like a3 | *Ipomoea batatas* | ACC61048 | 1,52E-30 |
| AU07-F03T7 | exonuclease family protein | *Lotus japonicus* | AFK41173 | 1,60E-39 |
| AU07-G08T7 | 60s ribosomal protein l11-2 | *Nicotiana tabacum* | CAC12883 | 1,42E-77 |
| AU07-G10T7 | two-component sensor histidine kinase | *Vitis vinífera* | XP_002267616 | 2,19E-05 |
| AU07-H01T7 | lysine histidine transporter 1 | *Vitis vinífera* | XP_002265308 | 3,98E-70 |
| AU07-H02T7 | phosphoribulokinase precursor | *Populus trichocarpa* | ABK95585 | 1,56E-51 |
| AU07-H05T7 | chlorophyll a-b binding protein | *Arabidopsis thaliana* | NP_198197 | 1,04E-31 |
| AU08-A06T7 | nad -binding rossmann-fold superfamily protein | *Ocimum basilicum* | AAX83110 | 7,23E-06 |
| AU08-B12T7 | probable e3 ubiquitin-protein ligase rnf217-like | *Populus trichocarpa* | XP_002336150 | 5,03E-10 |
| AU08-C09T7 | aspartic proteinase | *Solanum tuberosum* | AFX67029 | 1,17E-81 |
| AU08-E04T7 | cytochrome p450 family protein | *Olea europaea* | AFS28690 | 1,48E-62 |
| AU08-F03T7 | elongation factor 1 alpha | *Erythroxylum coca* | AEG78681 | 6,82E-85 |
| AU08-F08T7 | probable calcium-binding protein cml31 | *Datura metel* | AAM95458 | 1,78E-07 |
| AU08-G09T7 | 26s proteasome non-atpase regulatory subunit 2 homolog a-like | *Vitis vinifera* | XP_002277029 | 5,10E-105 |
| AU08-H12T7 | protein ethylene insensitive 3-like | *Nicotiana tabacum* | AAP03997 | 5,51E-43 |
| AU09-A01T7 | plastocyanin 1 | *Solanum lycopersicum* | P17340 | 4,14E-44 |
| AU09-A08T7 | 60s ribosomal protein l27a-3-like | *Vitis vinifera* | CBI34865 | 3,16E-09 |
| AU09-A10T7 | ankyrin repeat domain | *Nicotiana tabacum* | AAK18619 | 4,40E-45 |
| AU09-B03T7 | tubby-like f-box protein 8-like | *Medicago truncatula* | AFK44234 | 1,73E-41 |
| AU09-B10T7 | protein phloem protein 2-like a9 | *Populus trichocarpa* | XP_002321809 | 1,03E-03 |
| AU09-C10T7 | upf0326 protein at4g17486-like | *Populus trichocarpa* | XP_002324900 | 3,99E-26 |
| AU09-E03T7 | chlorophyll a b binding protein | *Ricinus communis* | XP_002531690 | 1,06E-139 |
| AU09-E04T7 | proline-rich protein | *Vitis vinifera* | XP_002272844 | 4,45E-62 |
| AU09-E09T7 | zinc finger | *Vitis vinifera* | XP_002265999 | 3,36E-58 |
| AU09-E11T7 | peptide transporter protein | *Ricinus communis* | XP_002518846 | 2,24E-09 |
| AU09-F06T7 | squamosa promoter-binding-like protein 1-like | *Vitis vinífera* | XP_003632418 | 8,21E-05 |
| AU09-F10T7 | sedoheptulose- -bisphosphatase | *Solanum lycopersicum* | NP_001234585 | 5,79E-115 |
| AU09-G05T7 | cytochrome p450 | *Medicago truncatula* | XP_003610581 | 9,24E-05 |
| AU09-H08T7 | stress-associated endoplasmic reticulum protein 2-like | *Medicago truncatula* | AFK38169 | 2,76E-05 |
| AU09-H10T7 | 60s ribosomal protein l7 | *Ricinus communis* | XP_002517516 | 2,05E-120 |
| AU10-A10T7 | protein chloroplastic-like | *Nicotiana benthamiana* | ABX26124 | 3,63E-08 |
| AU10-B02T7 | protein | *Vitis vinifera* | XP_003635272 | 2,18E-13 |
| AU10-B08T7 | peptide methionine sulfoxide reductase-like | *Solanum lycopersicum* | AEN03271 | 4,86E-52 |
| AU10-C11T7 | adenosylhomocysteinase 1 | *Scutellaria baicalensis* | AFV46215 | 8,40E-83 |
| AU10-D04T7 | 26s protease regulatory subunit 6b homolog | *Cicer arietinum* | CAA06853 | 1,91E-82 |
| AU10-D11T7 | probable nucleoredoxin 2-like | *Populus trichocarpa* | XP_002306954 | 2,09E-34 |
| AU10-E03T7 | kinase-like protein | *Medicago truncatula* | XP_003619232 | 2,21E-37 |
| AU10-E05T7 | upf0187 protein chloroplastic-like | *Vitis vinífera* | CBI40350 | 2,69E-74 |
| AU11-A01T7 | probable aldo-keto reductase 1-like | *Olea europaea* | ABS72001 | 4,17E-15 |
| AU11-D12T7 | adenosylhomocysteinase 1 | *Arabidopsis thaliana* | BAH57232 | 3,04E-110 |
| AU11-F06T7 | 14-3-3 protein | *Gossypium hirsutum* | ADK93080 | 2,14E-10 |
| AU11-G11T7 | 60s ribosomal protein l13a | *Hevea brasiliensis* | ADR71263 | 8,14E-12 |
| AU11-H05T7 | serine protease | *Solanum lycopersicum* | CAA07250 | 2,75E-10 |
| AU12-A03T7 | beta-galactosidase | *Cucumis melo* var. *cantalupensis* | BAH03319 | 4,01E-69 |
| AU12-A07T7 | transducin wd-40 repeat-containing protein | *Vitis vinífera* | XP_002282694 | 6,70E-40 |
| AU12-B05T7 | abhydrolase domain-containing protein fam108c1-like | *Populus trichocarpa* | XP_002329344 | 1,42E-16 |
| AU12-B11T7 | nucleoside diphosphate kinase | *Flaveria bidentis* | P47920 | 1,52E-44 |
| AU12-C05T7 | ubiquitin-like protein | *Morus mongólica* | AAZ82816 | 2,71E-85 |
| AU12-C08T7 | nucleoporin autopeptidase | *Glycine max* | XP_003539631 | 1,71E-31 |
| AU12-D02T7 | gdsl-motif lipase hydrolase | *Populus trichocarpa* | XP_002301596 | 2,79E-05 |
| AU12-D10T7 | 1-aminocyclopropane-1-carboxylate oxidase | *Plantago major* | CAH58646 | 1,03E-49 |
| AU12-F11T7 | act domain-containing protein | *Populus trichocarpa* | XP_002298369 | 4,11E-10 |
| AU12-H08T7 | 60s ribosomal protein l5-like | *Solanum melongena* | P93779 | 4,72E-18 |
| AU13-A01T7 | loxc homologue | *Olea europea* | ACD43485 | 1,81E-61 |
| AU13-A07T7 | Lipoxygenase 2 | *Olea europea* | ACD43484 | 8,73E-25 |
| AU13-B03T7 | chlorophyll a-b binding protein chloroplastic-like | *Gossypium hirsutum* | ACO51066 | 3,05E-51 |
| AU13-B08T7 | pinus taeda anonymous locus 0_17014_01 genomic sequence | *Ricinus communis* | XP_002528652 | 1,36E-22 |
| AU13-D10T7 | fiber protein fb15 | *Ricinus communis* | XP_002527804 | 5,20E-15 |
| AU13-D11T7 | nac domain ipr003441 | *Vitis vinifera* | XP_002284654 | 3,89E-06 |
| AU13-E01T7 | serine hydroxymethyltransferase 2 isoform 1 | *Olea europaea* | ABS72016 | 2,93E-86 |
| AU13-H10T7 | unnamed protein product | *Vitis vinifera* | CBI24541 | 5,49E-07 |
| AU14-C11T7 | photosystem i reaction center subunit n | *Ricinus communis* | XP_002526638 | 7,28E-44 |
| AU14-E01T7 | monoglyceride lipase-like isoform 2 | *Vitis vinifera* | XP_003635228 | 1,63E-05 |
| AU14-E11T7 | glutamine synthetase | *Arabidopsis thaliana* | BAD94507 | 6,22E-80 |
| AU14-F06T7 | gibberellin receptor | *Populus trichocarpa* | XP_002300570 | 4,59E-21 |
| AU14-G01T7 | pto kinase interactor | *Glycine max* | NP_001237620 | 1,11E-05 |
| AU-C1 | bark storage protein a-like | *Olea europaea* | AFP49328 | 3,56E-120 |
| AU-C2 | acid phosphatase | *Vitis vinifera* | CBI25273 | 6,74E-26 |
| AU-C4 | dolichyl-diphosphooligosaccharide--protein glycosyltransferase subunit stt3-like | *Vitis vinifera* | CAN67600 | 1,59E-22 |
| AU-C5 | photosystem ii reaction center w chloroplastic | *Populus trichocarpa* | XP_002300801 | 9,62E-32 |
| AU-C6 | calcium-dependent lipid-binding domain-containing protein | *Medicago truncatula* | ACJ84441 | 1,06E-13 |
| AU-C13 | protein | *Ricinus communis* | XP_002518215 | 2,28E-57 |
| AU-C14 | ferredoxin nadp+ partial | *Oryza sativa Indica* Group | ABR26035 | 2,25E-32 |
| AU-C19 | hypothetical protein VITISV_003407 | *Vitis vinifera* | CAN77337 | 5,10E-20 |
| AU-C21 | ac067971_24 ests gb | *Vitis vinifera* | CBI32019 | 2,27E-81 |
| AU-C26 | histone h3 | *Zea mays* | AFW71933 | 1,51E-41 |
| AU-C28 | alpha beta hydrolase fold superfamily | *Catharanthus roseus* | AAU95203 | 3,67E-59 |
| AU-C31 | protein | *Vitis vinifera* | CBI27372 | 3,00E-51 |
| AU-C35b | tubulin beta | *Ammopiptanthus mongolicus* | AFC01190 | 1,29E-31 |
| AU-C36 | heme-binding-like protein | *Vitis vinifera* | XP_002282544 | 5,44E-29 |
| AU-C38 | signal peptide peptidase-like 2b-like | *Glycine max* | XP_003552398 | 3,78E-61 |
| AU-C39 | photosystem ii oxygen-evolving complex protein 2 precursor | *Picea sitchensis* | ABR16937 | 1,98E-22 |
| AU-C41 | cytochrome b6-f complex iron-sulfur chloroplastic-like | *Vitis vinifera* | XP_002284361 | 1,14E-103 |
| AU-C42 | receptor-like protein kinase hsl1-like | *Populus trichocarpa* | XP_002328032 | 5,53E-55 |
| AU-C45 | nucleotide pyrophosphatase phosphodiesterase | *Cicer arietinum* | CAB71132 | 5,95E-80 |
| AU-C46 | 60s ribosomal protein l24 | *Populus trichocarpa* | XP_002305219 | 5,19E-15 |
| AU-C47 | psi reaction center subunit ii | *Vitis vinifera* | XP_002281825 | 1,23E-64 |
| AU-C51 | alpha beta hydrolase domain containing protein | *Vitis vinifera* | CAN63261 | 2,72E-08 |
| AU-C53 | heme-binding protein 2-like | *Glycine max* | XP_003517945 | 8,57E-68 |
| AU-C56 | protein executer chloroplastic-like | *Populus trichocarpa* | XP_002318040 | 1,09E-31 |
| AU-C58 | nascent polypeptide-associated complex subunit alpha-like protein 2-like | *Vitis vinifera* | XP_003634163 | 7,07E-44 |
| AU-C59 | gonadotropin beta chain | *Nicotiana tabacum* | AFK78108 | 5,28E-24 |
| AU-C66 | porin voltage-dependent anion-selective channel protein | *Solanum tuberosum* | P42055 | 1,31E-43 |
| AU-C69 | magnesium-dependent phosphatase | *Populus trichocarpa* | XP_002323938 | 1,34E-21 |
| AU-C70 | photosystem ii 10 kda polypeptide | *Vitis vinifera* | XP_002271791 | 5,28E-57 |
| AU-C71 | photosystem i reaction center subunit chloroplastic-like | *Phillyrea latifolia* | CAK18849 | 6,90E-65 |
| AU-C72 | senescence-associated protein | *Cupressus sempervirens* | ACA30301 | 4,79E-50 |
| AU-C74 | tcp-1 cpn60 chaperonin family protein | *Vitis vinifera* | XP_002284134 | 7,52E-64 |
| AU-C80 | bark storage protein a-like | *Hirudo medicinalis* | CCJ09766 | 9,17E-36 |
| AU-C85 | chaperone protein htpg family protein | *Arabidopsis thaliana* | BAF01597 | 6,19E-14 |
| AU-C94 | ras-related protein raba1f-like | *Vitis vinifera* | XP_002277579 | 3,37E-14 |
| AU-C96 | calmodulin | *Phaseolus vulgaris* | AAD10247 | 4,20E-32 |
| AU-C102 | kinase superfamily protein with octicosapeptide phox bem1p isoform 2 | synthetic construct | ABG54350 | 6,45E-113 |
| AU-C105 | high chlorophyll fluorescence phenotype 173 protein | *Ricinus communis* | XP_002513289 | 1,56E-47 |
| AU-C109 | superoxide dismutase | *Solanum tuberosum* | AAO16563 | 8,63E-18 |
| AU-C110 | wrky transcription factor 11-2 | *Medicago truncatula* | AFK39337 | 4,47E-16 |
| AU-C115 | bahd acyltransferase dcr-like | *Petunia* x *hybrida* | BAA93453 | 1,74E-87 |
| AU-C117 | metallothionein-like protein | *Lotus japonicus* | AFK33631 | 6,12E-18 |
| AU-C119 | 3-ketoacyl- thiolase | *Petunia x hybrida* | ACV70033 | 2,64E-66 |
| AU-C122 | cinnamyl alcohol dehydrogenase | *Plantago major* | CAJ43717 | 3,27E-62 |
| AU-C123 | heme binding protein | *Vitis vinifera* | XP_002277687 | 1,44E-16 |
| AU-C126 | lysosomal alpha-mannosidase | *Glycine max* | XP_003548859 | 5,01E-16 |
| AU-C129 | ccr4-associated factor | *Capsicum annuum* | ABG66307 | 1,44E-12 |
| AU-C134 | sigma factor sigb regulation protein rsbq | *Glycine max* | XP_003550024 | 4,28E-59 |
| AU-C137 | wd-40 repeat family protein | *Vitis vinifera* | CBI19139 | 1,75E-37 |
| AU-C141 | bax inhibitor 1 | *Nicotiana tabacum* | AAK73102 | 1,57E-17 |
| AU-C148 | protein | *Vitis vinifera* | XP_002264344 | 3,97E-36 |
| AU-C149 | glucose-6-phosphate isomerase | *Glycine max* | XP_003526535 | 4,01E-34 |
| AU-C151 | transcription factor jerf1 | *Vitis vinifera* | XP_002267008 | 1,78E-50 |
| AU-C161 | expansin-like protein precursor | *Eucalyptus globulus* | AAZ08316 | 2,93E-10 |
| AU-C150 | bark storage protein a-like | *Vitis vinifera* | CBI28039 | 1,25E-42 |
| AU-C159 | type iif peroxiredoxin | *Ipomoea batatas* | AAP42502 | 2,60E-104 |
| AU-C160b | short chain alcohol | *Ricinus communis* | XP_002523859 | 2,91E-68 |
| AU-C166 | mitochondrial-processing peptidase subunit alpha | *Vitis vinifera* | XP_002283310 | 9,04E-38 |
| AU-C170 | cc-nbs-lrr resistance protein | *Vitis vinifera* | CAN77665 | 7,05E-10 |
| AU-C171 | expansin-like b1 | *Vitis vinifera* | XP_002273896 | 1,13E-07 |
| AU-C172 | calcium-binding protein cml27 | *Olea europaea* | Q9M7R0 | 8,36E-60 |
| AU-C177 | metallothionein-like protein | *Corchorus olitorius* | ABS72197 | 1,01E-13 |
| AU-C186 | PREDICTED: uncharacterized protein LOC100245140 | *Vitis vinifera* | XP_002282384 | 4,89E-30 |
| AU-C187 | carotenoid cleavage dioxygenase 4 | *Osmanthus fragrans* | ABY60887 | 9,85E-60 |
| AU-C191 | prefoldin 1 | *Vitis vinifera* | XP_002264879 | 6,56E-51 |
| AU-C193 | protein | *Medicago truncatula* | AFK41421 | 1,68E-50 |
| AU-C200 | ubiquitin carrier protein | *Brassica napus* | ACC38297 | 7,89E-61 |
| AU-C204 | protein thylakoid chloroplastic-like | *Vitis vinifera* | CBI28372 | 1,05E-54 |
| AU-C208 | 60s acidic ribosomal protein p0 | *Solanum tuberosum* | ABB29933 | 3,39E-06 |
| AU-C209 | like protein | *Populus trichocarpa* | XP_002314095 | 4,60E-10 |
| AU-C217 | at3g23920-like partial | *Nicotiana langsdorffii* x *Nicotiana sanderae* | AAY89374 | 3,78E-82 |
| AU-C222 | binding protein | *Populus trichocarpa* | XP_002310360 | 7,28E-44 |
