# Supplementary material for: The biocontrol endophytic bacterium Pseudomonas fluorescens PICF7 induces systemic defense responses in aerial tissues upon colonization of olive roots
Source: Front Microbiol. 2014 Sep 5;5:427. doi: 10.3389/fmicb.2014.00427 (PMC4155815; doi:10.3389/fmicb.2014.00427)
Supplement: Supplementary file 2 [file DataSheet2.DOCX]

| **Table S2**. List of contigs and their corresponding contiguous/overlapping ESTs.  The EST Sequence Name refers to the codes (User_IDs) found within the cDNA library. AU means Arbequina aerial tissues inducted gene and AU-C indicates Arbequina aerial tissues identified as part of a contig. T7 refers to the forward T7 universal primers used for sequencing. | |
| --- | --- |
| **Contig names used in this study** | **EST sequences names (User_IDs) as found in dbEST/dbGSS/dbSTS databases** |
| AU-C1 | AU01-E06T7, AU01-C11T7, AU06-D01T7, AU05-C09T7, AU01-A03T7, AU02-B06T7, AU01-E10T7, AU02-G04T7, AU02-D11T7, AU03-D11T7, AU02-C05T7, AU01-G03T7, AU01-D02T7, AU10-B03T7, AU05-H07T7, AU04-C04T7, AU12-A08T7 |
| AU-C2 | AU01-A04T7, AU09-E10T7 |
| AU-C4 | AU01-A07T7, AU01-A08T7, AU11-D01T7 |
| AU-C5 | AU01-A09T7, AU07-H03T7, AU13-F11T7, AU01-A11T7 |
| AU-C6 | AU01-A10T7, AU02-F10T7, AU08-C08T7, AU09-H09T7, AU10-D02T7 |
| AU-C7 | AU01-H09T7, AU01-A12T7, AU06-H09T7 |
| AU-C12 | AU03-E12T7, AU01-B07T7 |
| AU-C13 | AU02-F07T7, AU07-C12T7, AU10-H03T7, AU01-B08T7, AU07-E03T7 |
| AU-C14 | AU01-B09T7, AU01-C01, AU02-G07T7, AU02-H11T7, AU01-C09T7 |
| AU-C15 | AU07-C09T7, AU01-B10T7, AU07-B05T7 |
| AU-C19 | AU01-C05T7, AU04-C02T7 |
| AU-C20 | AU01-C07T7, AU10-A04T7, AU03-H05T7, AU05-E06T7, AU04-B09T7, AU05-D03T7, AU09-H02T7 |
| AU-C21 | AU01-C10T7, AU06-A03T7 |
| AU-C22 | AU01-C12T7, AU05-C01T7 |
| AU-C26 | AU01-D05T7, AU04-C11T7, AU09-C03T7 |
| AU-C28 | AU01-D07T7, AU13-F03T7 |
| AU-C31 | AU01-D10T7, AU08-D12T7 |
| AU-C32 | AU01-D11T7, AU03-E05T7, AU06-B07T7, AU11-G12T7, AU01-E04T7, AU14-F10T7 |
| AU-C35b | AU04-G02, AU03-C07 |
| AU-C36 | AU01-E08T7, AU03-G02T7 |
| AU-C38 | AU01-E12T7, AU02-B08T7 |
| AU-C39 | AU01-F02T7, AU11-C05T7 |
| AU-C40 | AU12-D09T7, AU01-F03T7, AU04-A11T7, AU14-G04T7 |
| AU-C41 | AU01-G04T7, AU10-F08T7, AU06-G07T7, AU11-D10T7, AU12-E04T7 |
| AU-C42 | AU01-F05T7, AU10-C12T7 |
| AU-C43 | AU05-E04T7, AU01-F06T7 |
| AU-C45 | AU01-F10T7, AU04-E04T7 |
| AU-C46 | AU10-A12T7, AU01-F12T7 |
| AU-C47 | AU12-D08T7, AU01-G01T7 |
| AU-C48 | AU01-G07T7, AU14-C10T7 |
| AU-C51 | AU01-G10T7, AU13-E06T7 |
| AU-C53 | AU01-H03T7, AU13-A06T7 |
| AU-C56 | AU01-H07T7, AU05-E11T7 |
| AU-C58 | AU01-H10T7, AU14-A06T7, AU13-C10T7, AU11-F05T7 |
| AU-C59 | AU08-E07T7, AU01-H11T7, AU02-G06T7, AU07-G06T7, AU14-B11T7 |
| AU-C62 | AU02-A04T7, AU05-E07T7, AU08-C11T7 |
| AU-C66 | AU02-A08T7, AU13-A04T7, AU14-F04T7, AU14-H12T7, AU11-B05T7 |
| AU-C69 | AU02-B03T7, AU04-G04T7, AU09-A06T7, AU14-E07T7 |
| AU-C70 | AU02-B05T7, AU11-F07T7, AU09-D11T7, AU08-H09T7 |
| AU-C71 | AU02-B07T7, AU05-G05T7, AU02-D08T7, AU10-F01T7 |
| AU-C72 | AU02-B09T7, AU13-F01T7 |
| AU-C74 | AU02-B11T7, AU02-D02T7 |
| AU-C80 | AU03-E01T7, AU02-C09T7, AU09-A12T7, AU03-H09T7, AU12-G01T7, AU08-B04T7, AU04-B05T7, AU11-B02T7 |
| AU-C85 | AU02-E02T7, AU10-F09T7 |
| AU-C86 | AU02-E03T7, AU14-B08T7 |
| AU-C94 | AU02-G02T7, AU09-A04T7 |
| AU-C96 | AU02-G05T7, AU04-A09T7, AU10-C03T7 |
| AU-C97 | AU02-G08T7, AU02-H05T7, AU05-B05T7, AU08-H07T7, AU12-H03T7, AU13-F02T7 |
| AU-C100 | AU10-B02T7, AU02-H08T7 |
| AU-C102 | AU02-H10T7, AU03-H07T7 |
| AU-C105 | AU03-A09T7, AU03-B08T7 |
| AU-C109 | AU07-C07T7, AU14-D11T7, AU03-B07T7, AU07-C04T7 |
| AU-C110 | AU03-B09T7, AU08-A05T7 |
| AU-C115 | AU05-E10T7, AU04-G11T7, AU03-D03T7, AU06-H10T7, AU10-D05T7 |
| AU-C117 | AU04-H07T7, AU03-D10T7, AU07-D10T7 |
| AU-C119 | AU04-F05T7, AU03-E08T7, AU08-E01T7 |
| AU-C122 | AU03-H11T7, AU03-F04T7 |
| AU-C123 | AU03-F05T7, AU04-D10T7 |
| AU-C126 | AU03-G10T7, AU11-A08T7 |
| AU-C127 | AU03-G12T7, AU06-D04T7 |
| AU-C129 | AU08-B03T7, AU09-H06T7, AU03-H03T7 |
| AU-C134 | AU07-F02T7, AU04-C07T7, AU04-A08T7 |
| AU-C135 | AU04-A12T7, AU08-C04T7, AU12-A10T7 |
| AU-C137 | AU04-B06T7, AU14-A08T7 |
| AU-C141 | AU04-D05T7, AU11-C11T7 |
| AU-C144 | AU08-H11T7, AU04-E07T7, AU06-E12T7 |
| AU-C148 | AU04-G08T7, AU09-H01T7 |
| AU-C149 | AU04-H05T7, AU08-B05T7 |
| AU-C150 | AU04-H06T7, AU12-C04T7 |
| AU-C151 | AU04-H08T7, AU07-H09T7 |
| AU-C152 | AU04-H09T7, AU11-F09T7 |
| AU-C157 | AU05-B04T7, AU06-G03T7, AU08-C03T7, AU08-G11T7 |
| AU-C159 | AU05-B10T7, AU06-C12T7 |
| AU-C160b | AU05-C03, AU13-B12 |
| AU-C161 | AU10-E09T7, AU05-C04T7 |
| AU-C166 | AU05-E02T7, AU06-B11T7 |
| AU-C168 | AU05-G06T7, AU06-G05T7, AU10-H11T7 |
| AU-C169 | AU05-G09T7, AU13-D04T7 |
| AU-C170 | AU05-H01T7, AU12-E01T7 |
| AU-C171 | AU05-H06T7, AU12-B03T7 |
| AU-C172 | AU05-H12T7, AU13-A09T7 |
| AU-C175 | AU06-B06T7, AU13-A03T7 |
| AU-C176 | AU06-B08T7, AU08-A04T7 |
| AU-C177 | AU06-B12T7, AU14-D12T7 |
| AU-C178 | AU06-C02T7, AU13-E03T7 |
| AU-C180 | AU08-G12T7, AU06-C11T7 |
| AU-C186 | AU06-E10T7, AU14-D02T7 |
| AU-C187 | AU07-C03T7, AU06-F05T7, AU14-F05T7 |
| AU-C191 | AU06-H03T7, AU08-F05T7, AU10-H02T7 |
| AU-C193 | AU06-H11T7, AU14-A01T7 |
| AU-C200 | AU07-C01T7, AU09-C02T7 |
| AU-C203 | AU07-F09T7, AU08-B06T7 |
| AU-C204 | AU07-F12T7, AU08-G06T7, AU11-C10T7, AU08-A07T7 |
| AU-C208 | AU07-H06T7, AU08-G10T7, AU10-C02T7 |
| AU-C209 | AU08-A11T7, AU09-E05T7 |
| AU-C217 | AU09-E07T7, AU09-F01T7 |
| AU-C222 | AU10-B12T7, AU14-B06T7 |
| AU-C228 | AU14-G09T7, AU11-G05T7 |
| AU-C232 | AU13-C03T7, AU13-H01T7 |
